# Epithelial Gab1 calibrates RIPK3-dependent necroptosis to prevent intestinal inflammation

**DOI:** 10.1172/jci.insight.162701

**Published:** 2023-03-22

**Authors:** Jiaqi Xu, Shihao Li, Wei Jin, Hui Zhou, Tingting Zhong, Xiaoqing Cheng, Yujuan Fu, Peng Xiao, Hongqiang Cheng, Di Wang, Yuehai Ke, Zhinong Jiang, Xue Zhang

**Affiliations:** 1 Department of Pathology, Sir Run Run Shaw Hospital;; 2 Department of Pathology and Pathophysiology, and Department of Respiratory Medicine of Sir Run Run Shaw Hospital;; 3 Department of General Surgery and; 4 Department of Gastroenterology, Sir Run Run Shaw Hospital;; 5 Department of Pathology and Pathophysiology, and Department of Cardiology of Sir Run Run Shaw Hospital; and; 6 Institute of Immunology and Sir Run Run Shaw Hospital, Zhejiang University School of Medicine, Hangzhou, China

**Keywords:** Gastroenterology, Inflammation, Inflammatory bowel disease, Molecular pathology, Signal transduction

## Abstract

As a hallmark of inflammatory bowel disease (IBD), elevated intestinal epithelial cell (IEC) death compromises the gut barrier, activating the inflammatory response and triggering more IEC death. However, the precise intracellular machinery that prevents IEC death and breaks this vicious feedback cycle remains largely unknown. Here, we report that Grb2-associated binder 1 (Gab1) expression is decreased in patients with IBD and inversely correlated with IBD severity. Gab1 deficiency in IECs accounted for the exacerbated colitis induced by dextran sodium sulfate owing to sensitizing IECs to receptor-interaction protein kinase 3–mediated (RIPK3-mediated) necroptosis, which irreversibly disrupted the homeostasis of the epithelial barrier and promoted intestinal inflammation. Mechanistically, Gab1 negatively regulated necroptosis signaling through inhibiting the formation of RIPK1/RIPK3 complex in response to TNF-α. Importantly, administration of RIPK3 inhibitor revealed a curative effect in epithelial Gab1-deficient mice. Further analysis indicated mice with Gab1 deletion were prone to inflammation-associated colorectal tumorigenesis. Collectively, our study defines a protective role for Gab1 in colitis and colitis-driven colorectal cancer by negatively regulating RIPK3-dependent necroptosis, which may serve as an important target to address necroptosis and intestinal inflammation–related disease.

## Graphical abstract

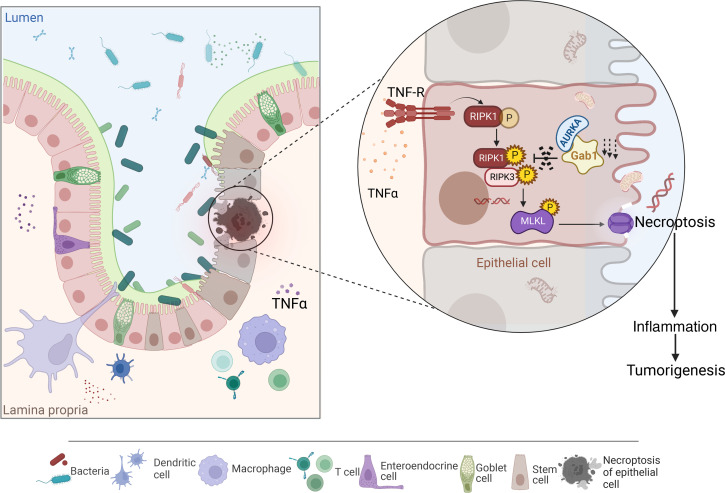

## Introduction

Inflammatory bowel disease (IBD), typically categorized as ulcerative colitis (UC) and Crohn’s disease (CD), is a group of chronic relapsing inflammatory disorders that affect the gastrointestinal tract in humans (1, 2). Recent reports highlight the central role of intestinal epithelial cells (IECs) in initiation and exacerbation of IBD (3–6). IECs linked by tight junctions serve as a physical and biochemical barrier, which facilitates host-microorganism interactions to orchestrate mucosal immunity ranging from tolerance to antipathogen response (7, 8). However, intestinal epithelial barrier breach leads to intestinal hyperpermeability and microbial invasion, followed by immune activation, cytokine release, and mucosal inflammation, which triggers more IEC death and barrier defects, perpetuating further inflammation and thus forming a vicious cycle in IBD development (9, 10).

IECs undergo dynamic cell turnover, necessitating a tightly regulated mechanism between proliferation and cell death to safeguard intestinal barrier function, wherein aberrant cell death in intestinal epithelium is a hallmark both in IBD patients and in mouse colitis models (11, 12). Recently, regulated necrotic cell death, namely necroptosis, has been receiving emerging attention as a newly identified type of epithelial cell death regulating intestinal homeostasis and inflammation (13–15). Necroptosis is a form of lytic, nonapoptotic programmed cell death triggered by pro-death and innate immune signals, such as TNF-α, IFNs, and pathogen-associated molecular patterns (16, 17). The initiation of necroptosis involves the activation of protein kinases receptor-interaction protein kinase 1 and 3 (RIPK1 and RIPK3), which subsequently results in phosphorylation and oligomerization of mixed lineage kinase-like protein (MLKL) to assemble membrane pores (18), ultimately leading to the release of damage-associated molecular patterns (DAMPs), including IL-1α and alarmin high mobility group box 1 (HMGB1) (19–21). A number of genetic studies reveal that mice with genetic ablation of *Caspase-8*, Fas associated via death domain (*Fadd*), or SET domain bifurcated histone lysine methyltransferase 1 (*Setdb1*) in IECs develop spontaneous ileitis and colitis owing to sensitizing cells to RIPK3-mediated necroptosis (22–24). Moreover, clinical evidence of aberrant RIPK3 activation and necroptotic cell death has been reported in pediatric and adult IBDs (22, 25, 26). However, how IEC necroptosis is negatively regulated in IBD development remains elusive to date. Therefore, it is of great importance to dissect negative regulation of necroptosis to maintain intestinal barrier homeostasis and optimal immune response.

Grb2-associated binder 1 (Gab1) is an adaptor protein originally identified downstream of growth factor and cytokine receptors, including EGFR, insulin receptor, and c-Met (27, 28), and thus participates in governing diverse biological events, including cell proliferation, development, and angiogenesis. Recent reports demonstrate the essential role for Gab1 in maintaining lung surfactant homeostasis and regulating acute lung inflammation and chronic asthmatic inflammation (29, 30), as well as controlling tissue repair processes including pulmonary and liver fibrosis (31, 32). In addition, cardiac Gab1 deletion leads to heart failure in aged mice associated with cardiomyocytes’ apoptosis (33), in which apoptosis is considered immunologically silent and restricts the spread of inflammation (11). Nevertheless, the pathophysiological functions of epithelial Gab1 in cell death under conditions of intestinal inflammation remain largely unknown.

In this study, we demonstrated that Gab1 expression was strikingly decreased in patients with IBD as well as in a mouse colitis model. Deletion of Gab1 in epithelial cells rendered mice susceptible to dextran sodium sulfate–induced (DSS-induced) colitis by perpetuating RIPK3-dependent necroptosis. Consistently, administration of RIPK3 inhibitor showed significant alleviation of colitis in Gab1-deficient mice. Furthermore, epithelial Gab1-deficient mice displayed aggravated colorectal cancer driven by chronic colitis. Together, our findings underscore that Gab1 acts as a critical determinant for IEC necroptosis and intestinal inflammation and thus may provide new insights into a therapeutic strategy for IBD treatment.

## Results

### Gab1 expression is decreased in IBD.

**Figure 1:**
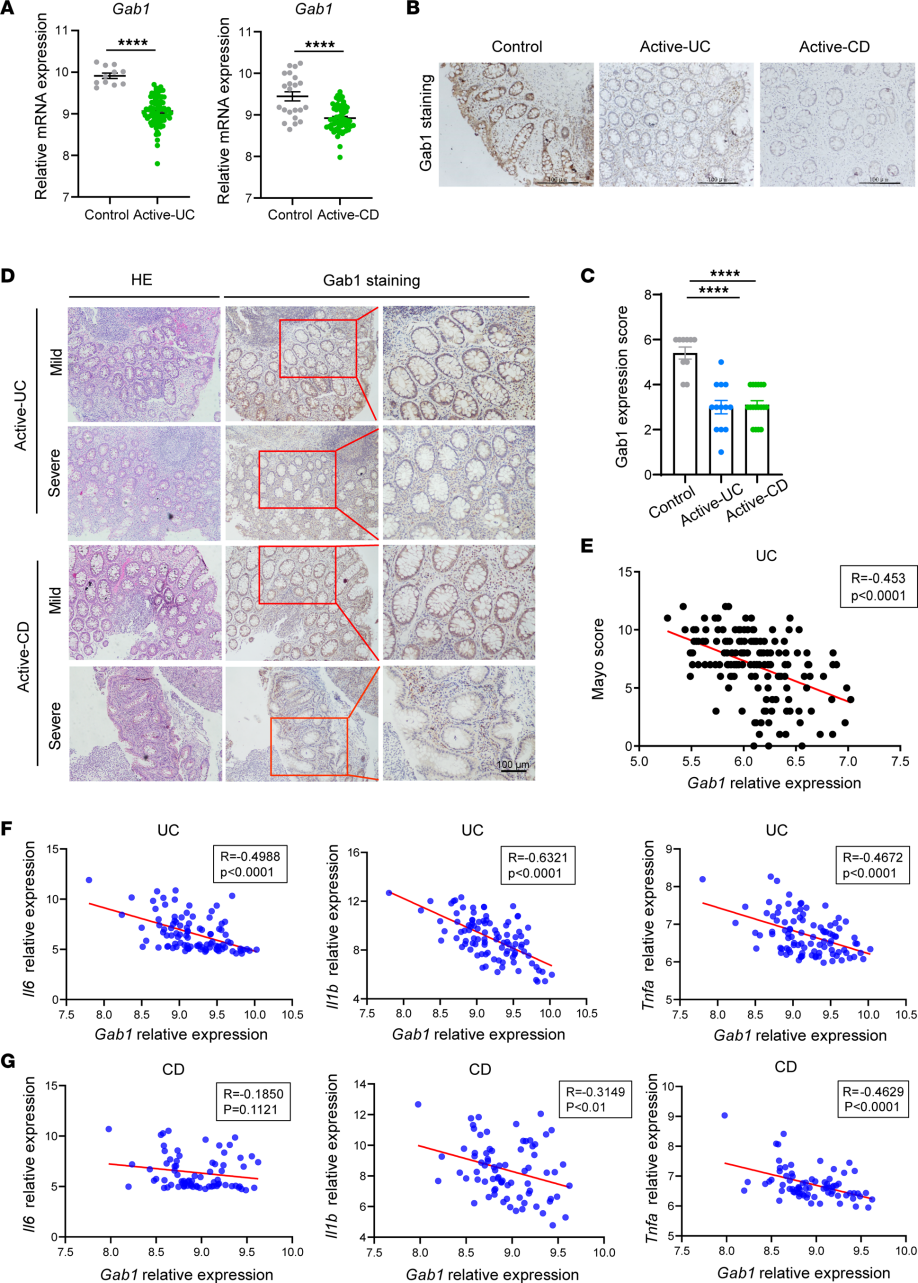
Decreased Gab1 expression in human IBD samples. (**A**) *Gab1* mRNA expression in patients with active UC (*n* = 74) and matched normal controls (*n* = 11), or active CD (*n* = 59) and matched normal controls (*n* = 22), revealed by analyzing a database of RNA-Seq data (GEO accession number GSE75214). (**B** and **C**) Representative IHC staining of Gab1 in colonic mucosa from patients with active UC (*n* = 13) or active CD (*n* = 18) and normal controls (*n* = 10). Scale bars, 100 μm. (**D**) Representative images showing hematoxylin-eosin (H&E) staining and Gab1 IHC staining in mild and severe IBD samples. *n* = 3 for each group. Scale bars, 100 μm. (**E**) Pearson’s correlation analysis was performed between relative *Gab1* expression and clinical Mayo score from UC patients (*n* = 162). *R* = –0.453, *P* < 0.0001. Data were collected from GEO database GSE92415. (**F**) Pearson’s correlation analysis between *Gab1* expression and pro-inflammatory cytokines *Il1b*, *Il6*, or *Tnfa* in colon biopsy samples from UC patients (*n* = 97). *R* = –0.4988, *P* < 0.0001 for *Il6*; *R* = –0.6321, *P* < 0.0001 for *Il1b*; *R* = –0.4672, *P* < 0.0001 for *Tnfa*. Data were collected from GEO database GSE75214. (**G**) Pearson’s correlation analysis between *Gab1* expression and pro-inflammatory cytokines *Il1b*, *Il6*, or *Tnfa* in colon biopsy tissues from CD patients (*n* = 75). *R* = –0.1850, *P* = 0.1121 for *Il6*; *R* = –0.3149, *P* < 0.01 for *Il1b*; *R* = –0.4629, *P* < 0.0001 for *Tnfa*. Data were collected from GEO database GSE75214. Quantitative data were shown as mean ± SEM. Statistical significance was assessed by using 2-tailed Student’s *t* test (**A**), 1-way ANOVA with multiple comparisons test (**C**), and Pearson’s correlation coefficient (**E**–**G**); ****P < 0.0001. UC, ulcerative colitis; CD, Crohn’s disease; GEO, Gene Expression Omnibus.

To establish the correlation of Gab1 with IBD progression, we analyzed a database of human samples from patients with UC or CD (34). The results showed that *Gab1* was dramatically downregulated in intestinal mucosa from patients with active UC and CD compared with the control group (Figure 1A), while *Gab1* was moderately decreased in UC and CD patients with remission (Supplemental Figure 1A; supplemental material available online with this article; https://doi.org/10.1172/jci.insight.162701DS1). Next, the reduction of Gab1 protein was confirmed in biopsy specimens from patients with active UC and CD by immunohistochemical (IHC) and immunofluorescence staining (Figure 1, B and C, and Supplemental Figure 1B). In contrast, the expression of homologous family protein Gab2 was comparable in the 3 groups of mucosal biopsies (Supplemental Figure 1C). Moreover, we found that Gab1 expression was markedly lower in patients with severe IBD than that in mild cases (Figure 1D). *Gab1* mRNA level was negatively correlated to the values of Mayo score in patients with UC (Figure 1E), and the expression of pro-inflammatory cytokines in patients with UC or CD (Figure 1, F and G), indicating that Gab1 was inversely correlated with IBD progression.

**Figure 2:**
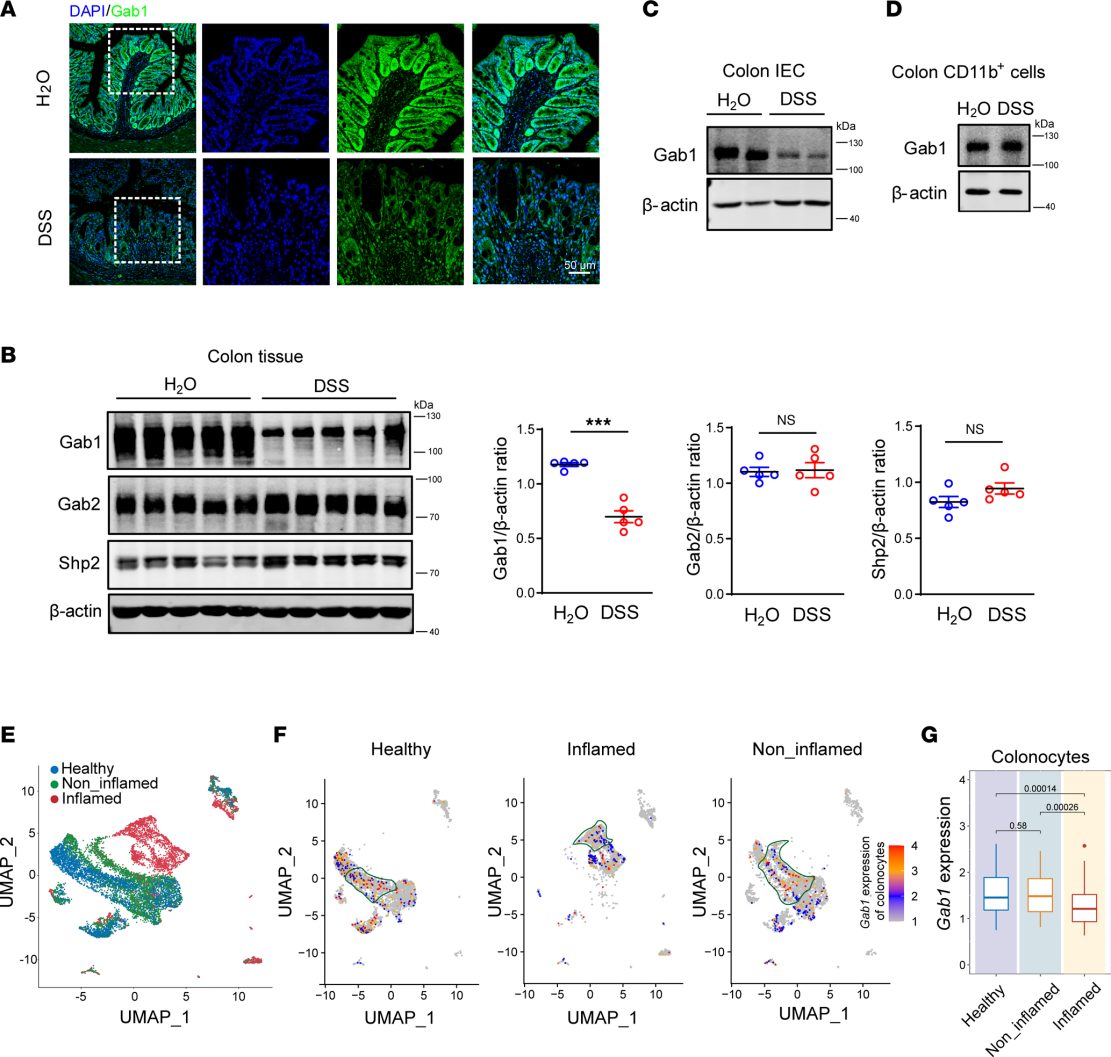
Gab1 is decreased in IECs during mouse colitis and human UC. (**A**) Representative immunofluorescence staining for Gab1 (green) and DAPI (blue) in mouse colons with or without a 7-day DSS treatment. Scale bars, 50 μm. (**B**) Western blotting of Gab1, Gab2, and Shp2 in mouse colonic tissues with or without DSS treatment. Quantitative analyses were determined on the right. *n* = 5 for each group. (**C**) Immunoblot analysis for Gab1 in IECs isolated from mouse colonic tissues treated as described above. *n* = 3 for each group. (**D**) Immunoblot analysis for Gab1 in CD11b^+^ cells sorted from colonic lamina propria (CLP) treated as described above. *n* = 3 for each group. (**E**) UMAP plots of single-cell clusters of colonic crypts from patients with inflamed/noninflamed UC and healthy controls (*n* = 3 per group) by analyzing a single-cell sequencing database (Gene Expression Omnibus [GEO] accession number GSE116222). (**F** and **G**) UMAP results (**F**) depicting *Gab1* expression and distribution mapped to referenced single-cell clusters (Supplemental Figure 2C), with box plot (**G**) demonstrating *Gab1* level in colonocytes of patients with inflamed/noninflamed UC and healthy controls (*n* = 3 per group) using the database as described above. Box plots show the interquartile range (box), median (line), and minimum and maximum (whiskers). Quantitative data were shown as mean ± SEM. Statistical significance was assessed by using 2-tailed Student’s *t* test (**B**) and Wilcoxon’s test (**G**); ***P < 0.001. DSS, dextran sodium sulfate; IEC, intestinal epithelial cell; UC, ulcerative colitis; UMAP, uniform manifold approximation and projection.

Similar to the observations in human IBD, immunofluorescence staining and Western blotting also revealed the decrease of Gab1 protein in DSS-induced colitis mouse model, while the expression of Gab2 and Gab1-binding protein Src homology region 2–containing protein tyrosine phosphatase 2 (Shp2) was unaltered (Figure 2, A and B). Next, IECs and CD11b^+^ myeloid cells were further insolated for immunoblotting. As shown in Figure 2, C and D, Gab1 was mainly decreased in IECs but not CD11b^+^ myeloid cells upon DSS treatment.

To further verify the reduction of Gab1 in human IECs, we utilized a single-cell RNA-sequencing data set of human colonic crypts from immunomodulatory treatment–naive patients with UC and healthy controls (35). The results revealed distinct cell cluster profiling in colonic crypts isolated from inflamed areas of patients with UC compared with healthy controls (Figure 2E and Supplemental Figure 2, A–C). UMAP plots showed differential distribution and apparent differences of Gab1 expression in these cell clusters (Figure 2F), in which Gab1 was predominantly decreased in colonocytes rather than other IEC subpopulations and immune cells in the inflamed group versus noninflamed and healthy controls (Figure 2, F and G, and Supplemental Figure 2D). These findings indicate that decreased Gab1 expression in IECs was correlated with intestinal inflammation in both humans and mice.

### Epithelial Gab1 deficiency renders mice susceptible to DSS-induced colitis.

To unravel the in vivo role of epithelial Gab1 in colitis, we first generated intestinal epithelium–specific *Gab1*-knockout (Gab1*^IEC^*-KO) mice. Conditional deletion of Gab1 was verified by genotyping and Western blot analysis (Supplemental Figure 3, A–C). These mice had no apparent gross phenotypes compared with their littermate controls. Also, no significant difference was found in the relative expression of mucins, antimicrobial peptides, or stem cell–associated genes in Gab1*^IEC^*-KO mice (Supplemental Figure 4).

**Figure 3:**
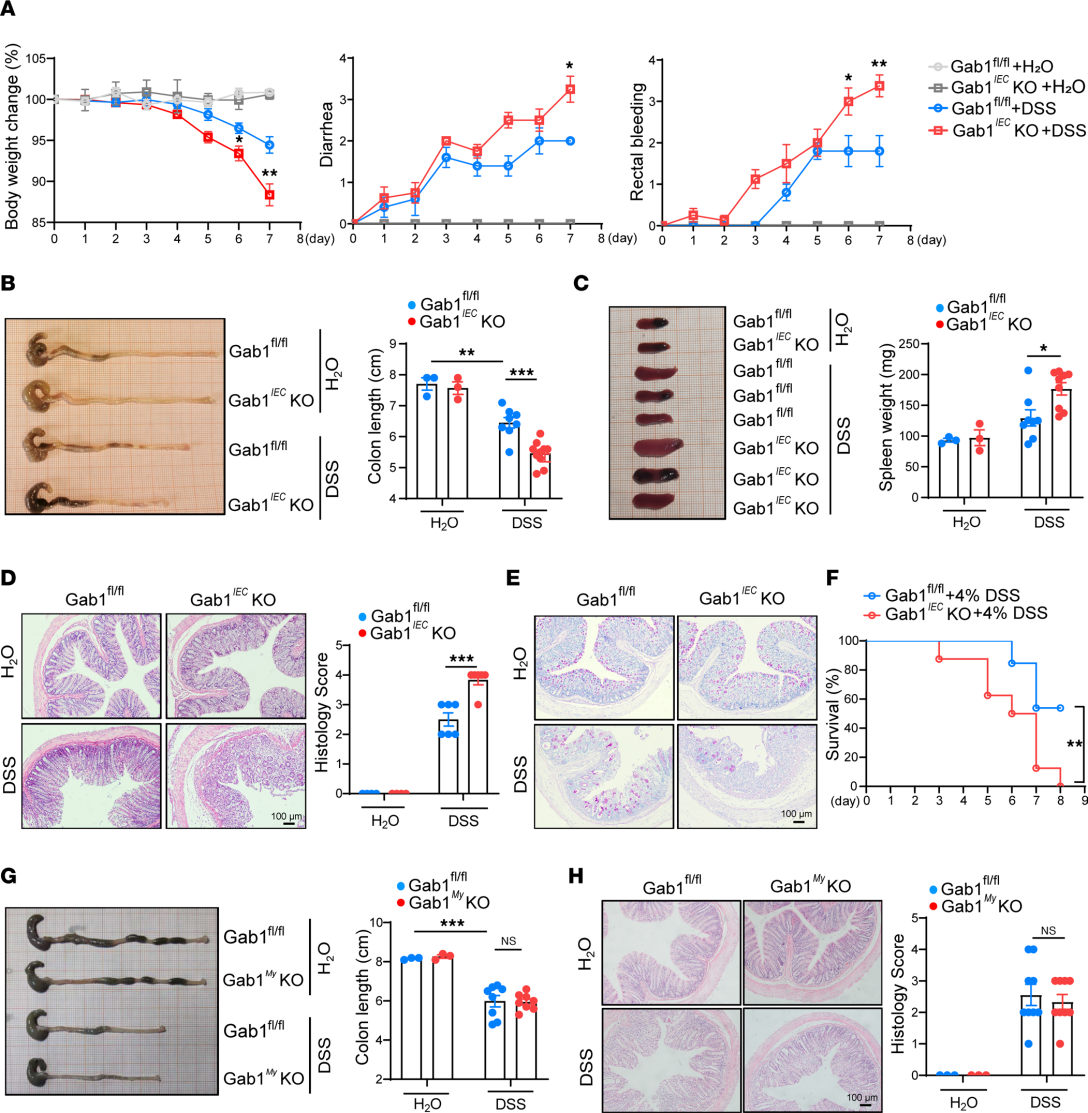
Epithelial Gab1 deficiency enhances susceptibility to DSS-induced experimental colitis. Gab1*^IEC^*-KO mice (**A**–**F**) or Gab1*^My^*-KO mice (**G** and **H**), as well as their littermates, were administrated with water or 3% DSS for 7 days to induce experimental colitis. (**A**) Body weight loss, diarrhea, and rectal bleeding were monitored daily with or without DSS treatment. *n* = 3, 3, 8, 9, for Gab1^fl/fl^ + H_2_O group, Gab1*^IEC^*-KO + H_2_O group, Gab1^fl/fl^ + DSS group, Gab1*^IEC^*-KO + DSS group, respectively. (**B**) Gross morphology images of the colon from Gab1^fl/fl^ or Gab1*^IEC^*-KO mice, and colon length were measured on day 7. *n* = 3, 3, 8, 9, respectively. (**C**) Gross morphology images of the spleen from Gab1^fl/fl^ or Gab1*^IEC^*-KO mice and spleen weight assessed on day 7. *n* = 3, 3, 8, 9, respectively. (**D**) Representative images of H&E-stained colons from Gab1*^IEC^*-KO mice and littermate controls, with histopathology analysis of colitis performed on day 7. Scale bars, 100 μm. *n* = 3, 3, 6, 6, respectively. (**E**) Representative periodic acid–Schiff (PAS) staining of colonic sections from Gab1*^IEC^*-KO mice and littermate controls. Scale bars, 100 μm. *n* = 3, 3, 6, 6, respectively. (**F**) Gab1^fl/fl^ (*n* = 13) and Gab1*^IEC^*-KO (*n* = 8) mice were challenged with 4% DSS for 7 days, and the survival of mice was monitored. (**G**) Gross morphology images of colons and colon length of Gab1^fl/fl^ and Gab1*^My^*-KO mice. *n* = 3, 3, 9, 9, respectively. (**H**) Representative H&E staining and histopathological scores of colonic sections from Gab1*^My^*-KO mice, as well as littermate controls. Scale bars, 100 μm. *n* = 3, 3, 9, 9, respectively. Quantitative data were shown as mean ± SEM and are representative of 3 independent experiments. Statistical significance was assessed by using 2-way ANOVA with multiple comparisons test (**A**–**D**, **G**, and **H**) and log-rank test (**F**); *P < 0.05, **P < 0.01, ***P < 0.001.

Then we induced colitis in the mouse model by administrating 3% DSS for 7 days. Compared with Gab1^fl/fl^ littermates, Gab1*^IEC^*-KO mice exhibited exacerbated colitis, as exemplified by more severe weight loss, diarrhea, and rectal bleeding (Figure 3A), as well as enhanced colon shortening and spleen swelling on day 7 when they were sacrificed (Figure 3, B and C). Consistent with these findings, histology analysis revealed that epithelial Gab1 deficiency led to more extensive disruption of the mucosal epithelium and inflammatory infiltration triggered by DSS (Figure 3D). Also, there was a decrease in the number of goblet cells in Gab1*^IEC^*-KO colonic epithelium compared with controls upon DSS treatment (Figure 3E). When challenged with 4% DSS, Gab1*^IEC^*-KO mice manifested significantly diminished survival rate compared with Gab1^fl/fl^ mice (Figure 3F).

To further verify the impact of myeloid Gab1 deficiency on colitis development, myeloid-specific *Gab1*-knockout (Gab1*^My^*-KO) mice were subsequently generated (Supplemental Figure 3, D and E). Gab1*^My^*-KO mice and their littermates were also subjected to DSS treatment as described above. Upon DSS induction, Gab1*^My^*-KO mice displayed comparable colitis-induced macroscopic changes (Figure 3G and Supplemental Figure 5) and histopathological damage with Gab1^fl/fl^ mice (Figure 3H). Taken together, these findings define a predominant role of Gab1 in IECs, rather than in myeloid cells, in protecting mice from DSS-induced colitis.

### Gab1 deficiency in IECs exacerbates inflammatory responses.

**Figure 4:**
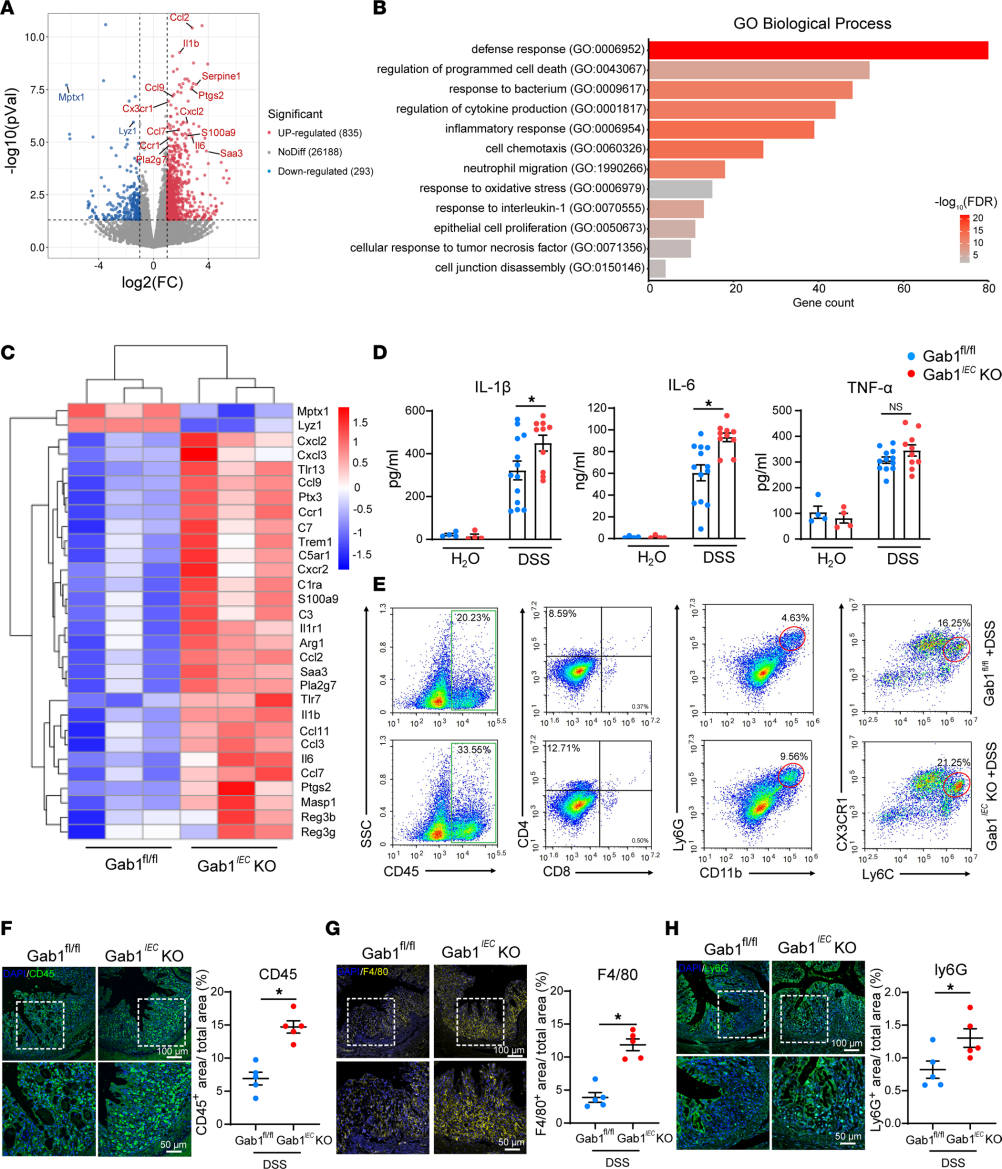
Loss of Gab1 in IECs exacerbates inflammatory response in vivo. Gab1^fl/fl^ and Gab1*^IEC^*-KO mice were challenged with 3% DSS and sacrificed at day 7. (**A**) Volcano plot of RNA-Seq transcriptome data displaying the pattern of gene expression values for epithelial Gab1-deficient colons relative to littermate controls after DSS treatment. Red and blue dots, significantly up- and downregulated genes (*P* < 0.05, |log_2_FC| > 1). *n* = 3 for each group. (**B**) GO enrichment analysis classifying DEGs into biological process groups. (**C**) Clustered heatmap showing expression changes of inflammation-associated genes in epithelial Gab1-deficient colons versus littermate controls. Red strip, high relative expression; blue strip, low relative expression. *n* = 3 for each group. (**D**) Soluble cytokine levels in supernatant of cultured colonic tissue isolated from Gab1^fl/fl^ and Gab1*^IEC^*-KO mice with or without DSS treatment. *n* = 4, 4, 13, 10, for Gab1^fl/fl^ + H_2_O group, Gab1*^IEC^*-KO + H_2_O group, Gab1^fl/fl^ + DSS group, Gab1*^IEC^*-KO + DSS group, respectively. (**E**) Representative flow cytometry plots of colon-infiltrated immune cells isolated from CLP at day 7. *n* = 3 for each group. (**F**–**H**) Representative immunofluorescence images stained for CD45 (green) (**F**), F4/80 (yellow) (**G**), Ly6G (green) (**H**), and DAPI (blue) from colon section with quantifications shown on the right. Scale bars, 100 μm (overview) and 50 μm (magnification). *n* = 5 for each group. Quantitative data were shown as mean ± SEM. Statistical significance was assessed by using 2-way ANOVA with multiple comparisons test (**D**) and 2-tailed Student’s *t* test (**F**–**H**); *P < 0.05. GO, Gene Ontology; DEGs, differentially expressed genes; CLP, colonic lamina propria; SSC, side scatter.

To gain insight into the changes in biological processes and pathways caused by Gab1 deletion, we subsequently performed RNA-sequencing (RNA-Seq) analysis using colon tissues obtained from Gab1*^IEC^*-KO and Gab1^fl/fl^ mice after a 7-day DSS treatment. A total of 1,128 differentially expressed genes (DEGs), including 835 upregulated and 293 downregulated genes, were shown as volcano plots compared with the controls (Figure 4A). Moreover, Gene Ontology (GO) analysis for biological processes highlighted that DEGs were most enriched in inflammation-related terms, programmed cell death, and cell junction disassembly (Figure 4B). Meanwhile, a clustered heatmap diagram illustrated that Gab1-deficient colons presented a pronounced bowel-inflammatory signature with high expression levels of IBD-related cytokines, chemokines, and inflammatory markers including *Il1b*, *Il6*, *Cxcl2*, and *Saa3* (Figure 4C). The transcriptome sequencing data were further validated by quantitative PCR (qPCR) and ELISA. Consistent with RNA-Seq results, the expression of pro-inflammatory cytokines, chemokines, and antimicrobial peptides was elevated in Gab1-deficient colon tissues (Supplemental Figure 6). A substantial increase of IL-1β and IL-6 protein was also observed in Gab1-deficient colonic supernatant (Figure 4D). Similarly, immunofluorescence staining showed obviously increased accumulation of CD45^+^ immune cells, F4/80^+^ macrophages, and Ly6G^+^ neutrophils in colonic sections of epithelial Gab1-deficient mice (Figure 4, F–H). Also, FACS supported the significant increase in the frequencies of colon-infiltrating immune cells (CD45^+^), neutrophils (CD11b^+^Ly6G^+^), and inflammatory macrophages (Ly6C^hi^CX3CR1^int^) in Gab1*^IEC^*-KO mice, whereas the frequencies of CD4^+^ T cells and CD8^+^ T cells were similar between Gab1-deficient and control groups (Figure 4E and Supplemental Figure 7). Taken together, these results indicate that epithelial Gab1 deficiency facilitates inflammatory cell infiltration and aggravates colonic inflammation in a colitis microenvironment.

### Gab1 maintains intestinal epithelial integrity through restricting aberrant necroptosis.

**Figure 5:**
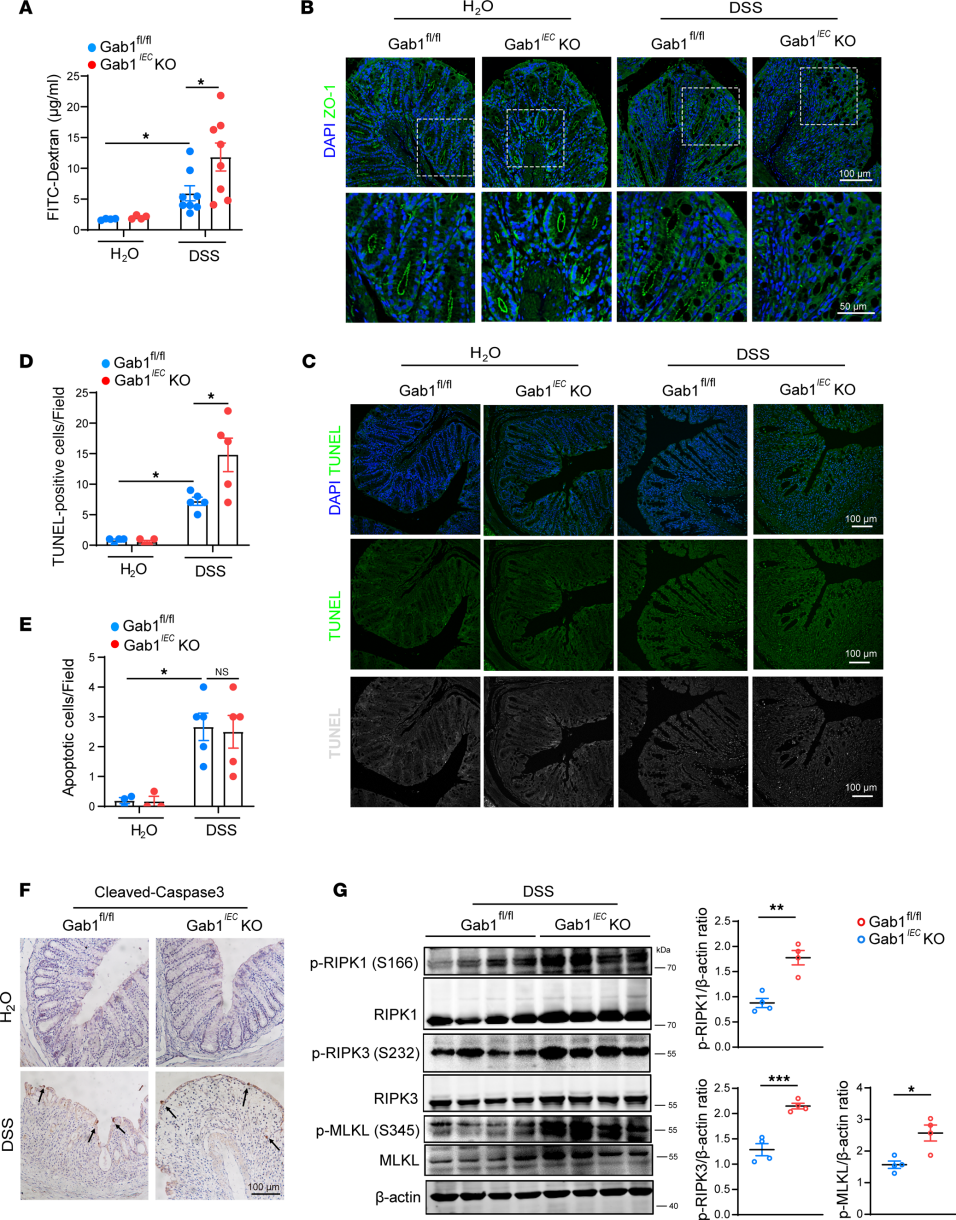
Gab1 maintains intestinal epithelial integrity by restricting aberrant necroptosis. (**A**) Gab1^fl/fl^ and Gab1*^IEC^*-KO mice were administrated with 3% DSS for 7 days and fed with FITC-dextran intragastrically 4 hours before sacrifice, then serum FITC-dextran level was detected by fluorescence spectrophotometer. *n* = 4, 4, 8, 9, for Gab1^fl/fl^ + H_2_O group, Gab1*^IEC^*-KO + H_2_O group, Gab1^fl/fl^ + DSS group, Gab1*^IEC^*-KO + DSS group, respectively. (**B**) Immunofluorescence staining of cell tight junction protein ZO-1 (green) and DAPI (blue) in colonic sections from the mice (*n* = 5 per group) treated with DSS for 5 days. Scale bars, 100 μm (overview) and 50 μm (magnification). (**C** and **D**) Representative images of TUNEL staining (green or gray) (**C**) and the quantifications of TUNEL^+^ IECs in colonic sections on day 7 (**D**). *n* = 4, 4, 5, 5, respectively. Scale bars, 100 μm. (**E** and **F**) Representative cleaved caspase-3 IHC staining (**F**) and the number of apoptotic cells quantified (**E**) in colonic sections on day 7. Arrows indicate cleaved caspase-3–positive cells. *n* = 3, 3, 5, 5, respectively. Scale bars, 100 μm. (**G**) Immunoblots of colonic lysates following DSS treatment for 7 days on p-RIPK1 (S166), p-RIPK3 (S232), or p-MLKL (S345) and quantitative analyses determined by ImageJ (NIH). *n* = 4 for each group. Quantitative data were represented as mean ± SEM. Statistical significance was assessed by using 2-way ANOVA with multiple comparisons test (**A**, **D**, and **E**) and 2-tailed Student’s *t* test (**G**); *P < 0.05, **P < 0.01, ***P < 0.001.

It has been reported that patients with IBD and mice with experimental colitis experience an abnormal intestinal barrier (9, 35, 36). To investigate if Gab1 affects intestinal barrier function in DSS-induced colitis, FITC-dextran concentration in the serum was determined on day 7 after DSS treatment. As shown in Figure 5A, Gab1*^IEC^*-KO mice displayed higher serum FITC-dextran concentrations compared with the controls upon DSS treatment, while 2 groups of mice displayed similar epithelial permeability without DSS induction, indicating that Gab1 is required for maintaining mucosal permeability in an inflammatory environment. Tight junction protein ZO-1, an indicator of the colonic epithelial integrity, localizes to the cell border in IECs in steady state (37). In line with increased FITC-dextran permeability, we also observed considerable loss and compromised organization of ZO-1 in Gab1-deficient epithelium following DSS treatment (Figure 5B).

Dysregulated cell death in intestinal epithelium leads to epithelial barrier breach, dysbiosis, and systemic spread of pathogen, which is commonly observed both in patients with IBD and in preclinical colitis models (11, 38). Moreover, our GO analysis data underscored the critical role of Gab1 in the regulation of DEGs involved in programmed cell death (Figure 4B). Hence, we evaluated IEC death in this model by the TUNEL assay, which detects apoptosis and other types of cell death (39). Compared with Gab1^fl/fl^ littermates, TUNEL-positive epithelial cells were dramatically increased in Gab1-KO crypts challenged by DSS (Figure 5, C and D). By contrast, there was a minimal number of cleaved caspase-3–positive epithelial cells upon DSS treatment, and no significant difference was found between the 2 groups of mice (Figure 5, E and F). Also, immunoblotting assay revealed that the level of apoptosis-related Bcl-2 family proteins (including Bcl-2, Bcl-XL, and Bax), as well as caspase-3 activation, were not changed in Gab1*^IEC^*-KO mice challenged with DSS (Supplemental Figure 8A), ruling out a significant contribution of apoptosis in this model. Meanwhile, no apparent difference was observed in ferroptosis-related proteins between 2 groups of mice after DSS treatment, including ACSL4, GPX4, and FTH1 (Supplemental Figure 8B), suggesting that ferroptosis contributed minimally to the aggravated colitis in Gab1*^IEC^*-KO mice. In addition, the cleavage of GSDMD protein was comparable in the colons of 2 groups following DSS challenge (Supplemental Figure 8, C and D), implying the minor contribution of GSDMD-mediated pyroptosis in our context. Interestingly, colonic protein isolated from Gab1*^IEC^*-KO mice showed robust phosphorylation of RIPK1, RIPK3, and MLKL compared with the Gab1^fl/fl^ group (Figure 5G), indicating Gab1-deficient IECs undergo enhanced necroptosis during DSS treatment. Together, these data suggest that IEC necroptosis triggered by Gab1 deficiency aggravates intestinal barrier dysfunction and bowel inflammation in DSS-induced colitis.

### Gab1 deficiency promotes IEC necroptosis and aggravates intestinal inflammation.

Necroptosis is a lytic pro-inflammatory mode of cell death in which effector caspases are inhibited or inactive in response to TNF-α (40). To verify the mouse phenotype owing to enhanced necroptosis in Gab1*^IEC^*-KO mice was controlled by TNF-α, we performed TNF blockade assay utilizing infliximab (IFX) as previously described (41–43). The aggravated colitis due to epithelial Gab1 deficiency was rescued by IFX administration, as exemplified by significantly improved macroscopic changes, colon length, as well as intestinal epithelial integrity (Supplemental Figure 9, A, B, and D). Histology analysis also revealed that Gab1*^IEC^*-KO mice displayed approximate epithelial damage and inflammatory infiltration compared with Gab1^fl/fl^ mice after TNF neutralization (Supplemental Figure 9C).

**Figure 6:**
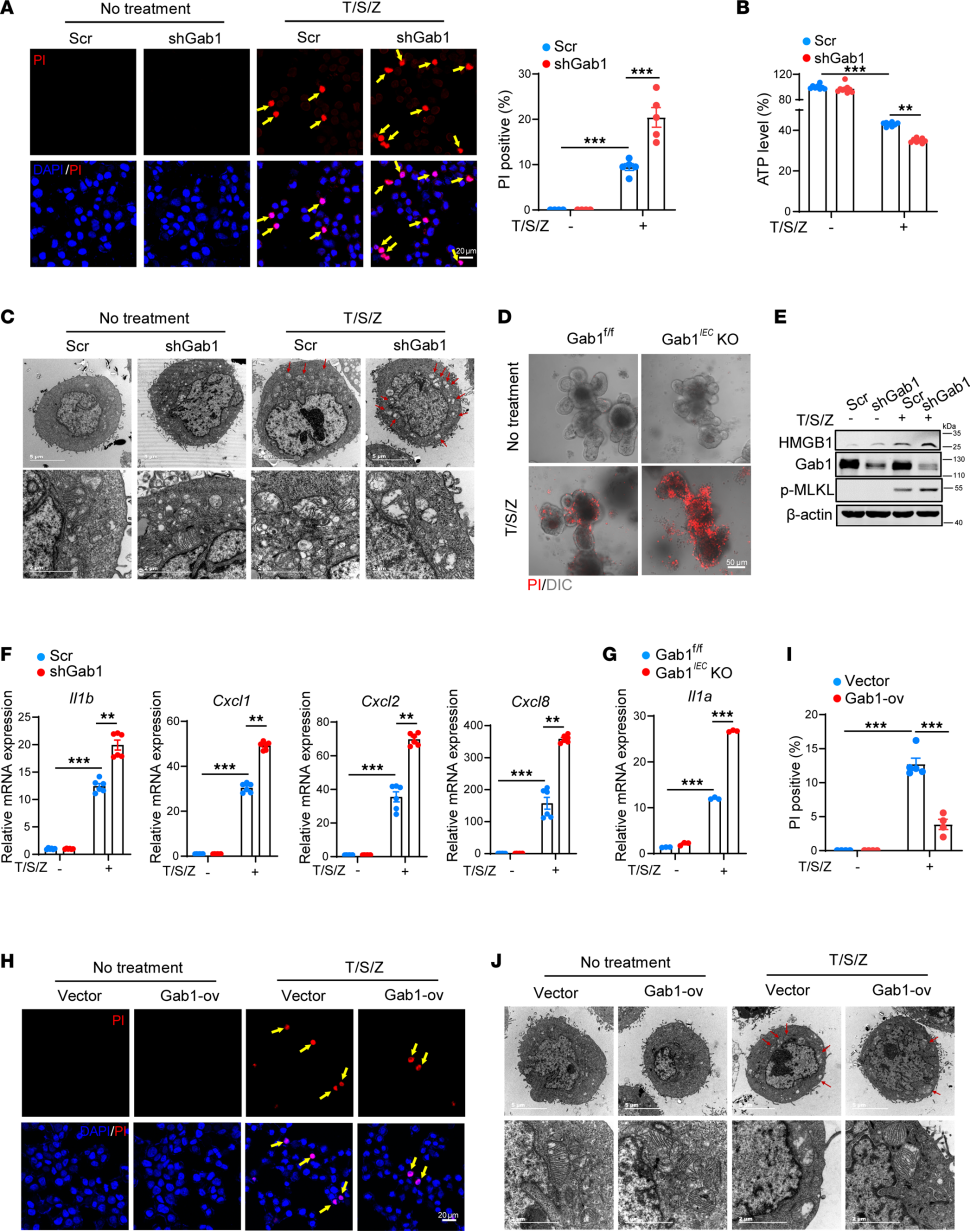
Gab1 deficiency sensitizes IECs to T/S/Z-induced necroptosis and inflammation. (**A**) Control (Scr) and Gab1-knockdown (shGab1) HT29 cells were treated with (*n* = 5) or without (*n* = 4) necrotic stimulation T/S/Z for 4 hours, then stained with propidium iodide (PI) (red) and DAPI (blue). Scale bars, 20 μm. Arrows indicate the cells undergoing necroptosis. T/S/Z, TNF-α (50 ng/mL), SM-164 (50 ng/mL), and Z-VAD-FMK (50 μM). (**B**) Viability of HT29 cells challenged with T/S/Z was determined by intracellular ATP levels using CellTiter-Glo assays. *n* = 8, 9, 8, 8, for Scr group, shGab1 group, Scr+T/S/Z group, shGab1+T/S/Z group, respectively. (**C**) Transmission electron microscopy (TEM) images showing the typical morphology of necroptosis in control (Scr) and Gab1-knockdown (shGab1) HT29 cells after T/S/Z treatment. Arrows point to the swelling mitochondria. Scale bars, 5 μm (overview) and 2 μm (magnification). (**D**) Representative confocal microscopy images of PI-stained intestinal organoids from Gab1^fl/fl^ and Gab1*^IEC^*-KO mice treated with T/S/Z for 8 hours. Scale bars, 50 μm. (**E**) Western blot displaying the release of HMGB1 protein in the supernatant from control (Scr) and Gab1-knockdown (shGab1) HT29 cells treated with T/S/Z. Gab1, p-MLKL (S358), and β-actin were immunoblotted in cell lysates, respectively. (**F**) Quantitative mRNA expression of *Il1b*, *Cxcl1*, *Cxcl2* and *Cxcl8* in control (Scr) and Gab1-knockdown (shGab1) HT29 cells upon T/S/Z treatment. *n* = 6 for each group. (**G**) Quantitative mRNA expression of *Il1a* in intestinal organoids from Gab1^fl/fl^ and Gab1*^IEC^*-KO mice upon T/S/Z treatment. *n* = 3 for each group. (**H** and **I**) HT29 cells were transfected with pXJ40-Flag vector or pXJ40-Gab1-Flag followed by T/S/Z stimulation as in **A**, then stained with PI (red) and DAPI (blue). Scale bars, 20 μm. Arrows indicate the cells undergoing necroptosis. *n* = 4, 4, 5, 4, respectively. (**J**) Representative TEM images for the typical morphology of necroptosis in control (Vector) and Gab1-overexpressed (Gab1-ov) HT29 cells. Arrows indicate the swelling mitochondria. Scale bars, 5 μm (overview) and 2 μm (magnification). Data were represented as mean ± SEM. All samples were biologically independent and 3 or more independent experiments were performed. Statistical significance was performed by using 2-way ANOVA with multiple comparisons test (**A**, **B**, **F**, **G**, and **I**); **P < 0.01, ***P < 0.001.

Next, we used HT29 cell line treated with a combination of T/S/Z [TNF-α (T), SM-164 (S), and pan-caspase inhibitor Z-VAD-FMK (Z)], which is a well-established model to study necroptosis in vitro. Immunofluorescence imaging showed a significant increase of propidium iodide–positive (PI-positive) necroptotic cells in Gab1-knockdown (shGab1) HT29 cells upon T/S/Z treatment (Figure 6A). Furthermore, cell viability determined by intercellular ATP was considerably decreased in shGab1 cells treated with T/S/Z (Figure 6B). Transmission electron microscopy (TEM) images also revealed exacerbated subcellular features of necrosis-like swelling in mitochondria challenged with T/S/Z in shGab1 cells (Figure 6C). Intestinal epithelium death was further reproduced ex vivo in the 3D mini-gut organoid culture based on previous research (14, 44). As shown in Figure 6D, PI staining revealed a remarkable increase of necroptotic cells in Gab1*^IEC^*-KO mouse–derived intestinal organoids following T/S/Z treatment. It has been highlighted that extensive necroptosis in IECs triggers intestinal inflammation by a massive release of DAMPs, which links cell death to mucosal inflammation (22, 45). Therefore, we examined the levels of DAMPs (including HMGB1, IL-1 family cytokines) as well as Cxcl family chemokines with or without Gab1. Western blotting revealed a higher amount of HMGB1 detectable in culture supernatant in Gab1-deficient cells after T/S/Z treatment (Figure 6E). qPCR analysis also displayed that Gab1 deletion resulted in significantly higher expression of *Il1a*, *Il1b*, *Cxcl1*, *Cxcl2*, and *Cxcl8* upon necroptosis stimulation (Figure 6, F and G). Consistent with above data, T/S/Z-triggered cell necroptosis was significantly restrained by the overexpression of Gab1, as indicated by both reduced PI-positive necroptotic cells (Figure 6, H and I) and mitigated swelling of mitochondria in HT29 cells (Figure 6J). Thus, these findings further support the role of Gab1 in negatively regulating epithelial necroptosis in response to TNF-α.

### Gab1 negatively regulates necroptosis through interacting with RIPK3 via aurora kinase A.

**Figure 7:**
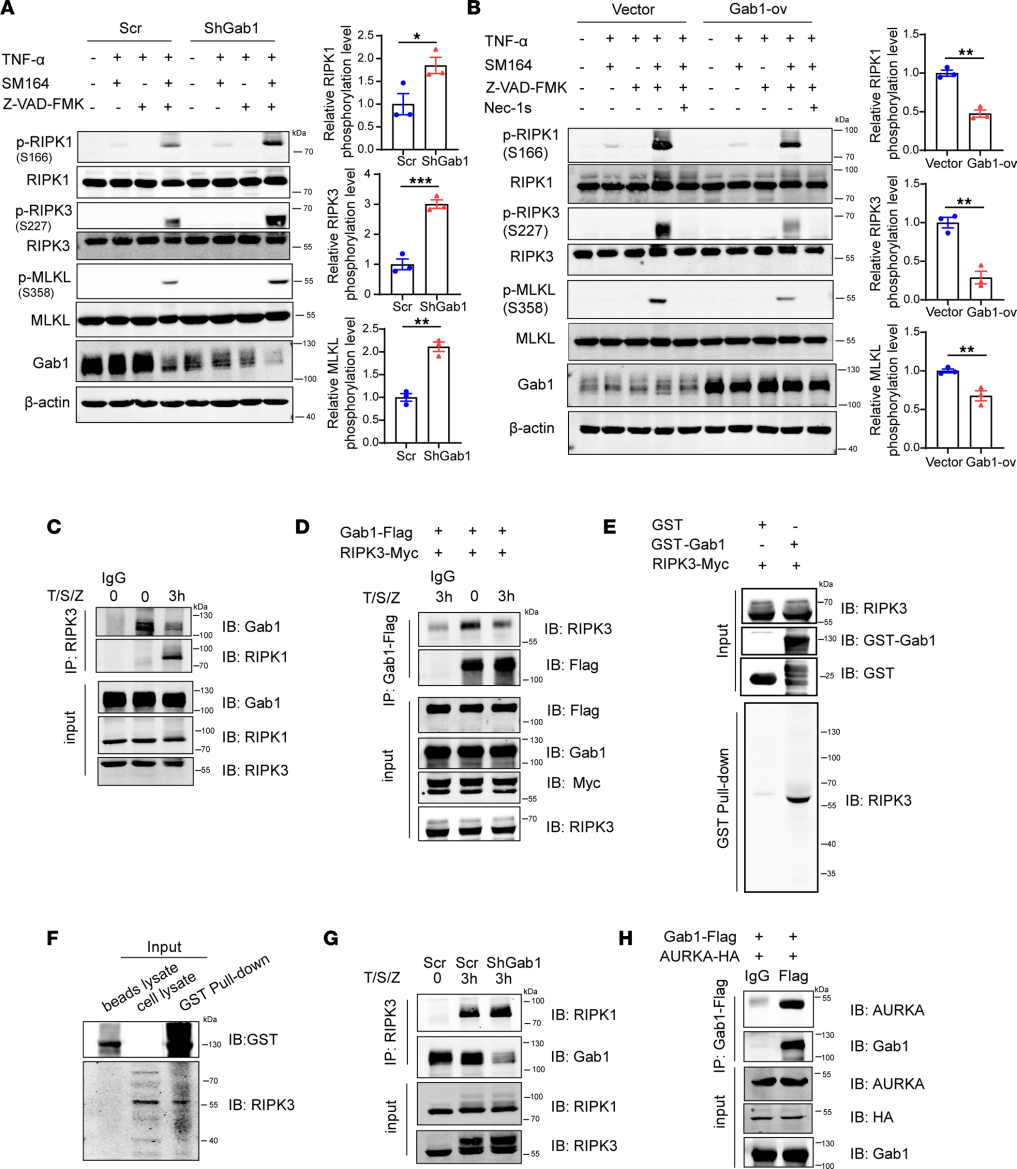
Gab1 blocks T/S/Z-induced necroptosis via binding with RIPK3. (**A**) Control or Gab1-knockdown HT29 cells were treated with DMSO, TNF-α+SM164, TNF-α+Z-VAD-FMK, or T/S/Z for 3 hours, and phosphorylation of RIPK1, RIPK3, and MLKL was determined by Western blotting. Quantitative data were shown as mean ± SEM for 3 independent experiments. (**B**) Control or Gab1-overexpressed HT29 cells were preincubated with 10 μm Nec-1s for 1 hours, followed by DMSO, TNF-α+SM164, TNF-α+Z-VAD-FMK or T/S/Z treatment for 3 hours. Detection of indicated proteins was carried out by Western blotting. Quantitative data were shown as mean ± SEM for 3 independent experiments. (**C**) HT29 cells were treated with T/S/Z for 3 hours. Total cell lysates were subjected to immunoprecipitation (IP) with anti-RIPK3 antibody or anti-IgG, followed by immunoblotting analysis with anti-Gab1 antibody or anti-RIPK1 antibody. See Supplemental Methods for antibody information and details on other methods. (**D**) HEK293T cells were co-transfected with Gab1-Flag and RIPK3-Myc for 24 hours, followed by T/S/Z stimulation for 3 hours. Cell lysates were then subjected to IP using anti-Flag antibody or anti-IgG and immunoblotted as indicated. (**E** and **F**) HEK293T cells were lysed and the supernatant was used to carry out a GST pull-down assay to detect the interaction between Gab1 and RIPK3. Recombinant GST-fused Gab1 protein was incubated with HEK293T cell lysates with (**E**) or without RIPK3 overexpression (**F**) and analyzed by immunoblotting with the anti-RIPK3 antibody. (**G**) Western blot showing co-IP assay for RIPK1 and RIPK3 interaction in control or Gab1-knockdown HT29 cells after exposure to T/S/Z for 3 hours. (**H**) HEK293T cells were cotransfected with Gab1-Flag and AURKA-HA for 24 hours. Total cell lysates were subjected to IP using anti-Flag antibody or anti-IgG, then immunoblotted with indicated antibodies. All samples were biologically independent and 3 independent experiments were performed. Statistical analysis was performed using 2-tailed Student’s *t* test; *P < 0.05, **P < 0.01, ***P < 0.001. AURKA, aurora kinase A.

Necroptosis signaling is mainly initiated by TNF-α, followed by the activation of protein kinases RIPK1 and RIPK3, which subsequently phosphorylate the downstream MLKL pseudokinase to induce cell membrane rupture and execute necroptosis (18, 46, 47). Western blot analysis showed that the phosphorylation level of RIPK1, RIPK3, and MLKL was remarkably elevated in Gab1-knockdown HT29 cells following T/S/Z treatment (Figure 7A), whereas overexpression of Gab1 suppressed T/S/Z-induced RIPK1/RIPK3/MLKL activation (Figure 7B). To dissect the underlying mechanism through which Gab1 negatively regulates the RIPK1/RIPK3/MLKL axis, we examined the interaction between Gab1 and pro-necroptotic factor RIPK3. As shown in Figure 7C, endogenous Gab1 bound with RIPK3 in basal state, and this association was impaired upon T/S/Z treatment in HT29 cells. Besides, similar effects were observed in HEK293T cells overexpressing Gab1-Flag and RIPK3-Myc plasmids, as demonstrated by co-IP (Figure 7D). The interaction of Gab1 and RIPK3 was further confirmed by a pull-down assay in vitro, as recombinant glutathione-*S*-transferase–fused (GST-fused) Gab1 protein was able to pull down RIPK3 from HEK293T cell lysates (Figure 7, E and F). Importantly, we found that Gab1 deficiency promoted the formation of RIPK1/RIPK3 complex under necroptotic conditions (Figure 7G) and thus facilitated downstream activation of necroptosis. Recent work showed that AURKA is a direct negative regulator of necrosome activation through binding the RIPK1/RIPK3 complex (48). Interestingly, AURKA was identified as a binding protein of Gab1 by using liquid chromatography with tandem mass spectrometry (Supplemental Figure 10) and confirmed by co-IP (Figure 7H). Thus, we propose that Gab1 interacts with RIPK3 through AURKA, thus restraining the assembly of RIPK1/RIPK3 complex. Collectively, these data indicate that Gab1 negatively regulates cell necroptosis by serving as a brake on RIPK1/RIPK3 complex formation in response to necroptotic signals.

### RIPK3 inhibition alleviates colitis in epithelial Gab1-deficient mice.

**Figure 8:**
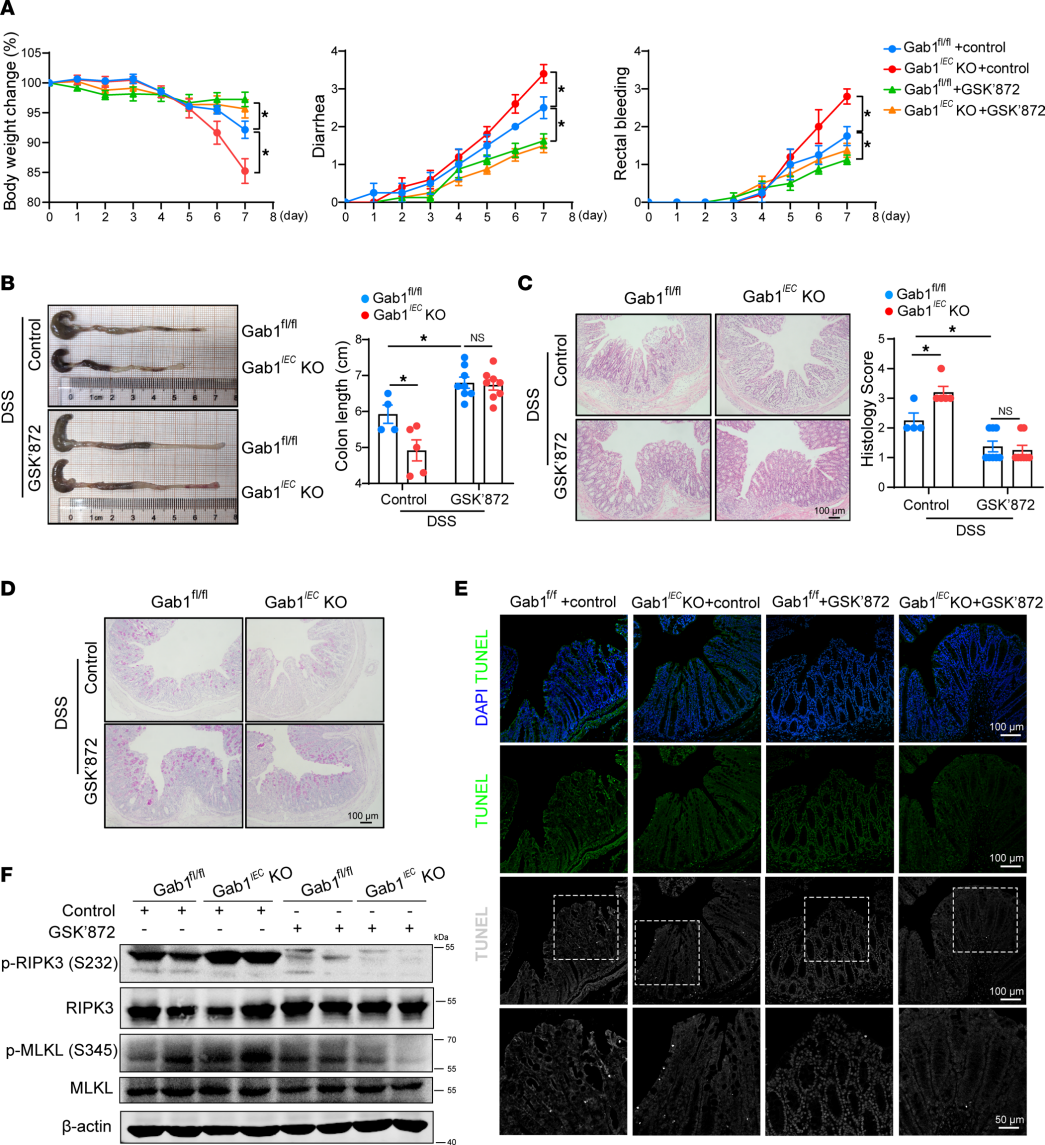
Selective RIPK3 inhibitor GSK’872 rescues epithelial Gab1-deficient mice from DSS-induced colitis. Gab1^fl/fl^ and Gab1*^IEC^*-KO mice were exposed to 3% DSS in drinking water as previously described, then intraperitoneally (i.p.) treated with either the vehicle control or GSK’872 at a dose of 10 mg/kg body weight once daily throughout the entire experimental period. *n* = 4, 5, 8, 8, for Gab1^fl/fl^ + DSS + control group, Gab1*^IEC^*-KO +DSS + control group, Gab1^fl/fl^ + DSS+ GSK’872 group, Gab1*^IEC^*-KO + DSS + GSK’872 group, respectively. (**A**) Relative percentage change in body weight, as well as diarrhea and rectal bleeding scores, were assessed daily. (**B**) Gross morphology images of the colon from Gab1^fl/fl^ and Gab1*^IEC^*-KO mice with different treatment. The colon lengths were measured on day 7. (**C**) Representative H&E-stained images and histological scores of the distal colon were assessed on day 7. Scale bars, 100 μm. (**D**) Representative PAS staining of colon sections from mice sacrificed at day 7. Scale bars, 100 μm. (**E**) Representative images of TUNEL staining (green or gray) of colonic sections on day 7. Scale bars, 100 μm (overview) and 50 μm (magnification). (**F**) Immunoblotting of colonic protein from the DSS-challenged mice with or without GSK’872 administration on day 7. Quantitative data were presented as the mean ± SEM. Statistical analysis was performed by 2-way ANOVA with multiple comparisons test; *P < 0.05.

We have shown that *Gab1* ablation in IECs contributed to the aggravated colitis due to excessive RIPK3-dependent necroptosis. Therefore, we utilized GSK’872, a specific RIPK3 inhibitor (49, 50), to further validate whether uncontrolled necroptosis in Gab1*^IEC^*-KO mice accounts for this phenotype. Gab1^fl/fl^ or Gab1*^IEC^*-KO mice were subjected to a 7-day course of 3% DSS and treated with either vehicle control or GSK’872 intraperitoneally. The results revealed that GSK’872 treatment showed curative effects in both Gab1^fl/fl^ and Gab1*^IEC^*-KO mice after DSS exposure. The aggravated colitis due to epithelial Gab1 deficiency was significantly rescued by GSK’872 administration, as exemplified by DSS-induced macroscopic changes (body weight loss, diarrhea, and rectal bleeding) being significantly alleviated (Figure 8A). Consistently, Gab1*^IEC^*-KO mice sacrificed on day 7 displayed approximate colon length (Figure 8B), epithelial damage, inflammatory infiltration (Figure 8C), and number of goblet cells (Figure 8D) compared with Gab1^fl/fl^ littermates after GSK’872 administration. Moreover, GSK’872 treatment substantially reduced the number of TUNEL-positive epithelial cells and the phosphorylation of RIPK3 and MLKL in colon tissues triggered by DSS (Figure 8, E and F). Taken together, these data indicate that hyperactivation of RIPK3 and necroptosis in IECs is primarily responsible for the exacerbated colitis in Gab1*^IEC^*-KO mice.

### Gab1 expression is associated with UC therapeutic outcomes and colorectal cancer.

**Figure 9:**
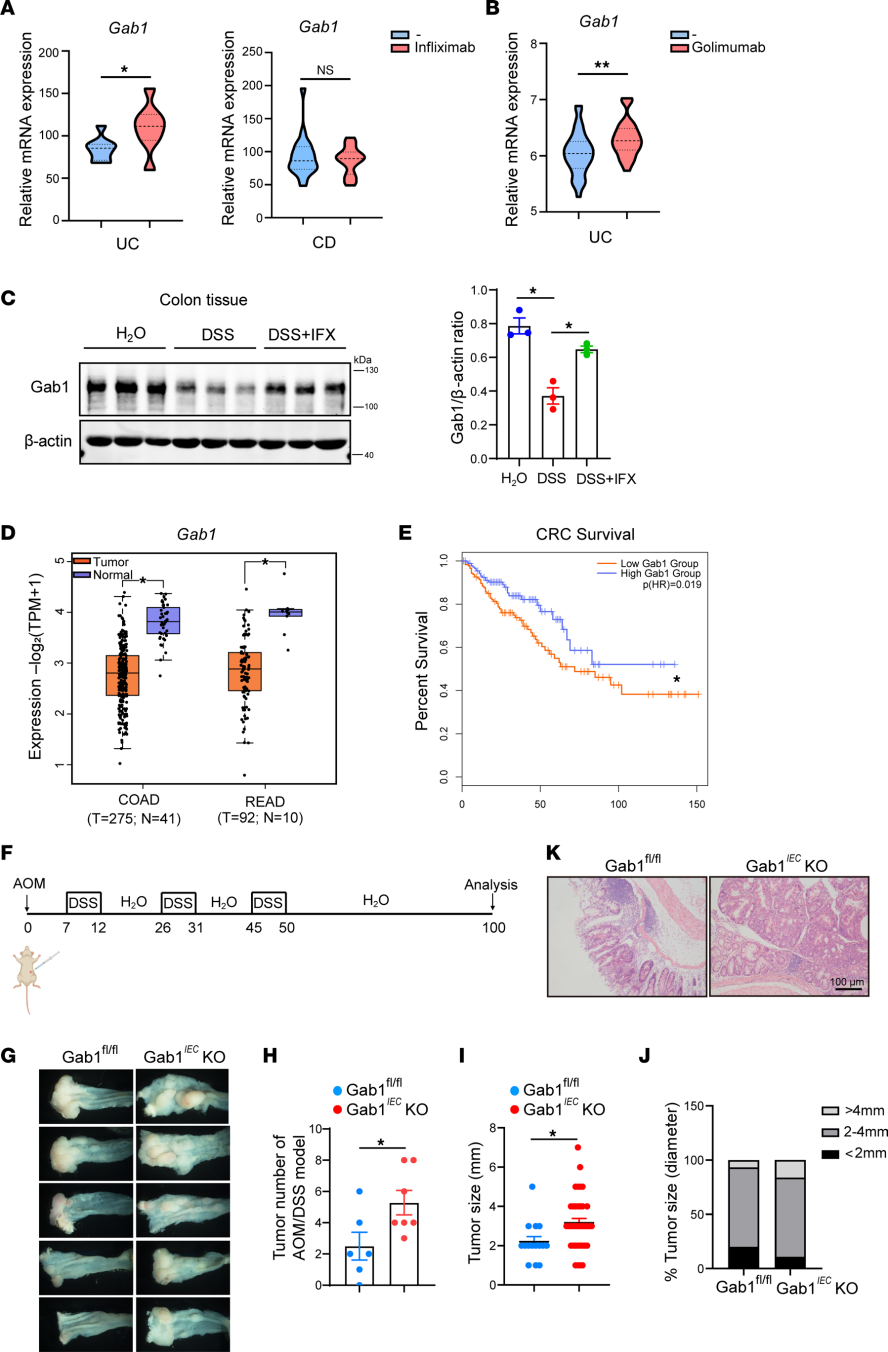
The clinical relevance of epithelial Gab1 in IBD treatment and CRC. (**A**) *Gab1* mRNA expression in colon biopsies from IFX-responded UC and CD patients before or after first IFX induction. The data were obtained from GEO database GSE16879. (**B**) *Gab1* mRNA expression in colon biopsies from golimumab-responded UC patients before or after first golimumab induction. The data were obtained from GEO database GSE92415. (**C**) Mice were subjected to a 7-day course of 3% DSS and treated with IFX intraperitoneally at day 5. Gab1 expression in colonic tissues was determined at day 10 by Western blotting, with quantifications shown on the right. *n* = 3 for each group. (**D**) Relative expression of *Gab1* in CRC (including COAD and READ) and matched normal tissue samples from GEPIA-based TCGA database. Box plots show the interquartile range (box), median (line), and minimum and maximum (whiskers). (**E**) Kaplan-Meier plots of patients with CRC with high or low *Gab1* expression; the data were collected from GEPIA-based TCGA database. (**F**) Schematic model of AOM/DSS-induced colitis-associated CRC (CAC). (**G**–**J**) Colorectal tumors were photographed (**G**) and the number of tumors per mouse (**H**), tumor size (**I**), as well as tumor diameter (**J**) were measured for each group. *n* = 6, 7, respectively. (**K**) Representative H&E staining of colonic sections from Gab1^fl/fl^ and Gab1*^IEC^*-KO mice. *n* = 6, 7, respectively. Scale bar, 100 μm. Data are shown as mean ± SEM; statistical analysis was performed using 2-tailed Student’s *t* test (**A**, **B**, **D**, **H**, and **I**) and 1-way ANOVA with multiple comparisons test (**C**); *P < 0.05, **P < 0.01. COAD, colon adenocarcinoma; READ, rectal adenocarcinoma; CRC, colorectal cancer; TCGA, The Cancer Genome Atlas; AOM, azoxymethane.

The introduction of anti-TNF therapy in the treatment of IBD has significantly improved the disease outcome. However, approximately 10%–40% of patients do not respond to induction therapy (primary nonresponse) (51). Thus, we assessed the *Gab1* expression in patients before and after the first anti-TNF treatment. We found that *Gab1* was dramatically upregulated in the inflamed mucosa of anti-TNF–responded patients with UC after the first IFX or golimumab treatment whereas *Gab1* was comparable in IFX-responded patients with CD before and after treatment (Figure 9, A and B). Subsequently, we utilized IFX, the chimeric mouse-human monoclonal antibody, to neutralize TNF activity in mice. Consistent with the observations in human UC, we found that the reduction of Gab1 expression in colonic tissues was significantly rescued after anti-TNF treatment in the DSS-induced colitis model (Figure 9C). These results suggested that the increase of *Gab1* expression after treatment may predict efficient therapeutic outcomes for UC.

Necroptosis is linked to inflammation, and chronic inflammation is suggested to be a high risk factor for colorectal cancer (CRC) (15, 49). Therefore, we analyzed the CRC database and found a significant decrease of *Gab1* expression in both patients with colon adenocarcinoma (COAD) and patients with rectal adenocarcinoma (READ) compared with healthy controls (Figure 9D). Moreover, overall survival data indicated that lower Gab1 expression related to poorer clinical outcomes (Figure 9E). Next, we used the azoxymethane (AOM)/DSS model of colitis-associated cancer (CAC) to further determine the role of Gab1 in inflammation-related tumorigenesis in vivo (Figure 9F) (52). As shown in Figure 9, G–J, Gab1*^IEC^*-KO mice exhibited a significantly increased number and size of tumors in the colorectum compared with Gab1^fl/fl^ mice. H&E staining showed larger tumors formed in epithelial Gab1-deficient colorectum (Figure 9K). These data suggest that Gab1 inhibits tumorigenesis driven by chronic colitis in mice and may serve as a tumor suppressor in human CRC.

In summary, our study defines a protective role for Gab1 in intestinal inflammation through restraining epithelial cell necroptosis.

## Discussion

Increasing evidence highlights that intestinal barrier dysfunction greatly contributes to the predisposition and perpetuation of IBD (53–55). However, the molecular mechanisms underlying the epithelial barrier maintenance remain obscure. Herein, we defined a crucial role for Gab1 in protecting intestinal barrier by preventing epithelial cell necroptosis during intestinal inflammation, which provides new insights into the diagnostic and therapeutic approach for IBD.

IECs, linked by tight junctions, form a permeable barrier separating luminal microbes and mucosal immune cells to coordinate an appropriate host response, ranging from tolerance to antipathogen immunity (7, 56). Serving as the first line of defense against microbial encroachment, impaired IEC function has been reported in a wide array of intestinal disorders, especially in IBD (9, 57, 58). Moreover, intestinal barrier dysfunction might precede the clinical diagnosis of IBD by years, invoking the possibility of preventing IBD through early and precise interventions (10). In this study, our data revealed that of Gab1 is remarkably decreased in both UC and CD patients, and the extent of reduction is negatively correlated with IBD progression. Single-cell RNA-Seq analysis of patients with UC, as well as a colitis mouse model, further identified that Gab1 is predominantly decreased in IECs, suggesting the probable role of epithelial Gab1 in disease progression.

Gab1 serves as an adaptor protein for integrating receptor-mediated signaling cascades downstream of growth factors or cytokines (27, 28). Global Gab1 ablation leads to embryonic lethality in mice, with profound developmental defects in heart, placenta, and skin (59). Besides the well-known function for Gab1, increasing evidence demonstrates that Gab1 participates in LPS-induced acute lung inflammation, house dust mite–induced asthmatic inflammation, and vascular inflammation, highlighting the unique role for Gab1 in inflammation-associated diseases (29, 30, 60). Furthermore, a recent study indicates that Gab family proteins Gab2/3 synergistically suppress colitis through controlling macrophage and CD8^+^ T cell activation (61). However, to date, the role of Gab1 in intestine and intestinal inflammation remains largely unknown. Considering the remarkable reduction of Gab1 in IECs we have demonstrated, VillinCre Gab1^fl/fl^ mice (Gab1*^IEC^*-KO) were first generated to dissect the role of Gab1 in intestinal inflammation. Notably, epithelial Gab1-deficient mice manifested exacerbated experimental colitis upon DSS treatment. Next, RNA-Seq data revealed excessive production of pro-inflammatory cytokines, chemokines, as well as antimicrobial peptides in Gab1*^IEC^*-KO colons. Further GO analysis underscored an enrichment of gene sets responsible for programmed cell death in the colonic transcriptome of Gab1*^IEC^*-KO mice, implicating the potential cellular process Gab1 involved during colitis development.

Epithelium homeostasis rests on the dynamic balance of cell proliferation and death, and aberrant increase of IEC death can lead to intestinal barrier disruption and inflammation. The regulation of cell death in IECs is highly context dependent. It has been reported that IECs undergo a series of cell death triggered by IBD, including apoptosis, necroptosis, pyroptosis, and ferroptosis (11, 14, 62–65). Recently, as a highly pro-inflammatory mode of cell death, necroptosis has emerged as a critical player in the modulation of intestinal homeostasis and inflammation that requires stringent control (15, 66, 67). Clinical evidence indicates necroptosis is highly active in both pediatric and adult IBDs (25, 26). Consistently, genetic approaches reveal a number of gene ablations, including of *Fadd*, *Casp-8*, and *Setdb1*, lead to spontaneous ileitis because of uncontrolled necroptosis but can be rescued on Ripk3^–/–^ or Mlkl^–/–^ (3, 22, 23). Several studies have linked Gab1 to cell death mainly through MAPK signaling. Cardiac Gab1 deletion leads to cardiomyocyte apoptosis and heart failure (33) while hepatocyte Gab1 controls the balance between acetaminophen-induced hepatocyte death and compensatory proliferation (68). Also, enhanced autophagy in Gab1-deficient vascular endothelial cells is observed in atherosclerosis. Here, we discovered a potentially novel role for Gab1 in regulating IEC necroptosis in a RIPK3-dependent manner. We found Gab1 deficiency rendered IECs susceptible to necroptosis, thereby shifting to aggravated barrier disruption and intestinal inflammation during DSS administration, whereas other types of cell death (apoptosis, pyroptosis, and ferroptosis) were unchanged. Collectively, we identified, for the first time to our knowledge, the novel protective role for Gab1 in restricting this lytic form of cell death during intestinal inflammation.

Different stimuli engage necroptosis, including TNF family, Toll-like receptors, and intracellular DNA/RNA sensors, with the best characterized being TNF-α (69). Given that the clinical efficacy of anti–TNF-α therapy has established TNF-α as a key player in IBD, we next focused on TNF-α–elicited necroptosis in our study. In response to TNF-α stimulation, RIPK1 can associate with RIPK3, leading to autophosphorylation and assembly of a necrosome complex, especially when caspase-8 activation is blocked or inefficient (40). RIPK3 subsequently phosphorylates the pore-forming protein MLKL to permeabilize the plasma membrane and release pro-inflammatory DAMPs, such as HMGB1 (19, 20, 70). To elucidate the mechanism of necroptosis affected by Gab1 deficiency, we used the human colorectal cell line HT29, which is a well-established cell line used to study the molecular machinery of necroptosis. Consistent with in vivo data above, we found pronounced induction of necroptotic cell death in the Gab1-KO group, as well as in Gab1-deficient intestinal organoids, while Gab1 overexpression suppressed necroptosis upon T/S/Z treatment. Mechanistically, Gab1 blocked assembly of RIPK1/RIPK3 complex depending on the ability of Gab1 to bind with RIPK3, thereby limiting the phosphorylation of RIPK3 and MLKL. Moreover, pharmacological inhibition of RIPK3 substantially alleviated experimental colitis with reduced inflammation in Gab1*^IEC^* -KO mice, suggesting that hyperactivation of RIPK3 and necroptosis largely contributes to exacerbated colitis in epithelial Gab1-deficient mice.

Chronic colitis is considered a high risk factor for CRC; therefore, we extended our studies to investigate the role for Gab1 in CAC and CRC. A previous study reported that Gab1 overexpression in DLD-1 colon carcinoma cells promoted tumor growth in a subcutaneous model (71), which is consistent with the in vitro findings from Bai et al. (72). In contrast, Liang et al. delineated the tumor-suppressing role for Gab1 in CRC (73). In our study, we generated mice with *Gab1*-KO in normal intestinal epithelium, followed by AOM/DSS induction to mimic the progression of inflammation-associated carcinogenesis. Gab1*^IEC^*-KO mice exhibited increased number and size of tumors in the colorectum compared with Gab1^fl/fl^ mice, suggesting a protective role for Gab1 in tumorigenesis driven by chronic colitis in mice, which is consistent with our bioinformatic results in patients with CRC.

Anti-TNF monoclonal antibodies have been extensively used for patients with IBD refractory to conventional medications such as corticosteroids and immunomodulators (74, 75). Effective treatment improves mucosal healing, and reduces hospitalizations and surgeries, but unfortunately, anti-TNF treatment failure is common. About 10%–40% of patients do not respond to induction therapy (primary nonresponse), and approximately 24%–46% of patients have secondary loss of response in the first year of treatment; 10% of patients have adverse drug effects (51). Our data revealed that *Gab1* was markedly elevated in mucosal biopsies obtained from patients with UC in response to IFX or golimumab, while the nonresponse group displayed minimal changes in *Gab1* expression, thus providing a genetic predictor to assess clinical outcomes of anti-TNF therapy in UC. However, there was only a slight rising trend of *Gab1* expression in inflamed mucosa before and after treatment in IFX-responded patients with CD. We speculate that this inconsistent change of *Gab1* expression occurred in responders with UC and CD possibly because of the underlying dissimilarities between UC and CD, while the considerable within-group differences due to the sample size of CD cohort may also account for this inconsistency. Currently, several RIPK1/RIPK3 inhibitors are now in clinical trials for the treatment of UC, expecting to prevent intestinal barrier breach by inhibiting IEC death and promoting resolution of inflammation (76, 77). Therefore, RIPK1/RIPK3 inhibitors may synergize with anti-TNF therapy to achieve higher immunosuppressive effects and improved outcomes.

In summary, our data demonstrate Gab1 as a critical regulator in maintaining epithelial barrier integrity to protect against intestinal inflammation through restricting aberrant necroptosis. These findings not only elucidate a deeper understanding of IBD pathogenesis by linking epithelial Gab1 with TNF-α–triggered necroptosis but also provide potentially new therapeutic strategies for personalized induction regimens to improve clinical outcomes more effectively.

## Methods

### Human samples.

Clinical samples of patients with UC and CD as well as healthy individuals were obtained from Sir Run Run Shaw Hospital, Zhejiang University School of Medicine, China. The diagnosis of UC or CD was based on clinical characteristics, endoscopic examination, histological analysis, and radiological criteria. Mayo Score and SES-CD were used to assess disease severity of the patients with UC and CD, respectively (78, 79). Healthy volunteers were recruited based on their medical history and routine laboratory tests including complete blood count and C-reactive protein. Normal ileal and colonic mucosa samples were obtained by endoscopy with the confirmation of endoscopic features and histological examination. The demographic information for the participants is detailed in Supplemental Table 1. The study was approved by the Medical Ethics Committee of Sir Run Run Shaw Hospital.

### Mice.

The Gab1^fl/fl^ mice are a gift from Gen-Sheng Feng (Department of Pathology, Division of Biological Sciences and Moores Cancer Center, University of California, San Diego, San Diego, California, USA). Gab1^fl/fl^ mice were crossed with Villin^Cre/+^ (GemPharmatech, C57BL/6 background) mice to generate IEC-conditional Gab1-knockout mice (VillinCre Gab1^fl/fl^, Gab1*^IEC^*-KO) and their littermate controls (Gab1^fl/fl^). Gab1^fl/fl^ mice were crossed with LysM^Cre/+^ (GemPharmatech, C57BL/6 background) mice to generate myeloid cell–specific Gab1-knockout mice (LysMCre Gab1^fl/fl^, Gab1*^My^*-KO) and their littermate controls (Gab1^fl/fl^). Mice were housed in a specific pathogen–free environment (25°C, suitable humidity, 12-hour light/12-hour dark cycle) and fed with sufficient water and food. All protocols of animal experiments were approved by the Animal Care and Use Committee of the Zhejiang University School of Medicine.

### Induction of colitis.

Mice were administrated with DSS in drinking water to induce colitis as previously described (52). In brief, 8- to 10-week-old Gab1–conditional KO mice and their littermates were treated with 3% DSS (MP Biomedicals) in drinking water (*w/v*) for 7 days and sacrificed for further study. During the experiment, body weights, diarrhea, and rectal bleeding were monitored daily. The scores were evaluated as follows: diarrhea: 0 = well-formed stools, 2 = soft and pasty stools, and 4 = watery stools; 0 = no bleeding, 2 = positive hemoccult, and 4 = gross bleeding.

### Single-cell RNA-Seq analysis.

Single-cell data used in this study were acquired from the GEO with accession number GSE116222 (35). A total of 11,175 cells from the colonic crypts of 3 patients with UC and 3 healthy individuals were downloaded as raw data for epithelial and immune cells. Data processing, including batch correction, doublet removal, gene annotation, and cell clustering, was performed as previously described. After that, the Seurat R package (version 2.3.2) was used to normalize expression values for total unique molecular identifier counts per cell. Cell clusters were visualized using a dimensionality reduction tool, namely UMAP. The statistical significance was assessed using Wilcoxon’s test.

### Cells.

HT29 cells were purchased from American Type Culture Collection (ATCC) and cultured in RPMI 1640 medium (Gibco) supplemented with 10% fetal bovine serum (FBS) (Gibco) along with 1% penicillin and streptomycin (HyClone) at 37°C and 5% CO_2_. HEK293T cells were purchased from ATCC and maintained in DMEM (Gibco) containing 10% FBS along with 1% penicillin and streptomycin at 37°C and 5% CO_2_. To construct Gab1-knockdown HT29 cell line, shGab1 cDNA were constructed into the PLKO1-puro vector (Addgene plasmid 8453) and packaged into lentiviruses. HT29 cells were infected with shGab1 lentivirus and then screened by puromycin.

For stimulation, cells were plated on 6-well plates at 5 × 10^5^ cells per well overnight and then pretreated with RIPK1 inhibitor Nec-1s (10 μM; Selleck Chemicals) for 1 hour. After that, cells were stimulated with T/S/Z mix — TNF-α, 50 ng/mL (Novoprotein); SM-164, 50 nM (Selleck Chemicals); Z-VAD-FMK, 50 μM (Selleck Chemicals) — for the indicated time. Cell lysates and culture supernatant were further analyzed by Western blot.

### GST fusion protein purification and pull-down assay.

Recombinant protein purification was performed as described previously (80). Briefly, the GST-tagged Gab1 full-length fusion proteins were expressed in *E*. *coli* BL21 cells (WEIDI, EC1002) at 16°C overnight to achieve maximal soluble expression. Cells were harvested and lysed by sonication in lysis buffer (20 mM Tris-HCl pH 7.5, 300 mM NaCl, 1% Triton X-100, and protease inhibitor cocktail [Roche, 04693132001]), then centrifuged at 12,000*g* for 15 minutes at 4°C. Supernatant was collected and incubated with glutathione-sepharose (GE Healthcare) overnight at 4°C. Total GST fusion protein concentration was estimated by using BCA Protein Assay kit (Beyotime). HEK293T cells were seeded onto 10 mm dishes transfected with full-length RIPK3 plasmids, then lysed with a lysis buffer (50 mM Tris-HCl, pH 7.4, 150 mM NaCl, 1% Triton X-100, 1% sodium deoxycholate, 0.1% SDS, 1 mM EDTA, and protease inhibitor cocktail). After centrifugation (4°C, 12,000*g*, for 15 minutes), cleared cell lysates were incubated with purified Gab1-bound glutathione-sepharose or control glutathione-sepharose overnight at 4°C with gentle rotation. Then the glutathione beads were washed 3 times with lysis buffer and eluted with loading buffer for Western blot analysis.

### Crypt isolation and organoid culture.

The intestinal organoids were derived from the small intestines as reported (24), with slight modifications. In brief, 10 cm small intestines were dissected and opened longitudinally to remove luminal contents. The intestine was cut into 5 mm pieces and incubated with 4 mM EDTA in PBS for 30 minutes at 4°C without shaking. Crypts were dissociated from villi by pipetting and filtered through a 70 μm strainer (BIOLGIX), followed by centrifugation (4°C, 200*g*, for 5 minutes) and washing. The purified crypts were resuspended in Matrigel (Corning, 356231) and seeded onto a glass-bottom dish, then cultured in IntestiCult Organoid Growth Medium (StemCell Technologies, 06005). Organoid growth medium was refreshed every 2–3 days. For the PI-traced organoid cell death assay, TNF-α (50 ng/mL), SM-164 (50 nM), and Z-VAD-FMK (50 μM) were added into organoid growth medium for 8 hours at day 7. Then organoids were stained with 1 μg/mL PI, and images were taken using a confocal microscope (FV3000, Olympus).

### Statistics.

Data are presented as mean ± SEM and statistical calculations were performed with GraphPad Prism 8.0. Statistical analysis was performed using 2-tailed unpaired Student’s *t* test (for 2-group comparison) or 1-way or 2-way ANOVA followed by Tukey’s multiple comparisons test (for multiple group comparison). *P* < 0.05 was considered statistically significant.

### Study approval.

For human samples, written informed consent was obtained from all individuals and all samples were deidentified. These studies were approved by the Medical Ethics Committee of Sir Run Run Shaw Hospital. All animal experiments were carried out in accordance with protocols approved by the Animal Care and Use Committee of the Zhejiang University School of Medicine.

## Author contributions

JX, ZJ, and XZ conceived of and designed the project. JX, SL, and WJ performed experiments. JX and SL analyzed and interpreted data. HZ and XC performed bioinformatic analyses. TZ and YF were in charge of recruiting patients/controls and collected tissue samples. JX, YK, and XZ wrote the manuscript and designed figures. PX, HC, DW, and ZJ edited the manuscript. All authors approved the final manuscript.

## Supplementary Material

Supplemental data

## Version 1 02/16/2023

In-Press Preview

## Version 2 03/22/2023

Electronic publication
