# Supplementary material for: Epithelial Gab1 calibrates RIPK3-dependent necroptosis to prevent intestinal inflammation
Source: JCI Insight. 2023 Mar 22;8(6):e162701. doi: 10.1172/jci.insight.162701 (PMC10070107; doi:10.1172/jci.insight.162701)
Supplement: Supplemental data [file jciinsight-8-162701-s050.pdf]

Supplemental Materials for

Epithelial Gab1 calibrates RIPK3-dependent necroptosis to prevent intestinal inflammation

Jiaqi Xu[1,7], Shihao Li[2,7], Wei Jin[3,7], Hui Zhou[2], Tingting Zhong[1], Xiaoqing Cheng[1], Yujuan Fu[1], Peng Xiao[4], Hongqiang Cheng[5], Di Wang[6], Yuehai Ke[2], Zhinong Jiang[1*] & Xue Zhang[2*]

*Correspondence to: zhangxue@zju.edu.cn or 3200039@zju.edu.cn.

This PDF file includes:

Supplemental Methods

Supplemental Figures 1 to 10

Supplemental Tables 1 to 6

Supplemental Methods

IHC staining and score

Human biopsy samples were fixed in 4% formalin, paraffin-embedded and cut into 4 μm sections. Then, paraffin sections were deparaffinized, rehydrated followed by antigen-retrieval in citric acid (10 mM, pH 6.0), and stained with primary antibody Gab1 (Abcam, ab59362) or Gab2 (Abcam, ab235932), and scored using Constantine's protocol. Briefly, Integrated staining intensity and the percentage of positive cells were semi-quantitatively. Staining intensity was evaluated as follows: 0 = no color; 1 = yellow; 2 = brown to yellow; and 3 = brown. The proportion of positive cells was scored as follows: 0 = positive cells < 10%; 1 = positive cells between 10% and 40%; 2 = positive cells between 40% and 70%; and 3 = positive cells ≥ 70%. The staining intensity score and proportion of positive cells score were then added up.

Isolation of colonic epithelial and immune cells

PBS-flushed colons were excised from control or $Gab1^{IEC}$ KO mouse longitudinally opened, and cut into 5mm pieces. Colon tissues were incubated with 10 ml HBSS supplemented with 5% FBS, 5 mM EDTA (Sigma-Aldrich) and 1 mM DTT (Sigma-Aldrich) at 37°C for 50min with shaking at 150 rpm to obtain epithelial cells for immunoblotting and flow cytometry analysis. The remaining tissues were flushed by PBS, cut into $1mm^2$ pieces, then digested with HBSS containing 5% FBS, 300 U/ml collagenase IV, and 5 U/ml DNaseI at 37°C for 45 min with shaking at 150 rpm. The lamina propria suspension was passed through a 70 μM sieve and washed with cold PBS to obtain single-cell suspension for the following FACS analysis and $CD11b^+$ cells isolation. Single-cell suspension from lamina propria was further labeled with Rat anti-CD11b antibody (ThermoFisher Scientific, MA1-10080) and incubated with Dynabeads® Sheep anti-Rat IgG (ThermoFisher Scientific) for 20min at 4 °C with gentle rotation. After that, the tube was placed in a magnet to remove the supernatant and resuspended in lysis buffer for protein extraction.

Flow cytometry

Single cells of lamina propria were prepared as described above and stained with anti-CD45-APC (eBioscience, 47-0451-82), anti-CD4-PE (BioLegend, 100407), anti-CD8-FITC (BioLegend, 100705), anti-Ly6G-PE (BioLegend, 127607), anti-CD11b-FITC (BioLegend, 101205), anti-CX3CR1-PE (BioLegend, 149006), anti-Ly6C-PE-Cy7 (BioLegend, 128017) according to manufacturer's protocols. The cells were analyzed with NovoCyteTM flow cytometer (ACEA). Compensation were performed between FITC and PE channel, as well as PE-Cy7 and PE channel, to correct for spectral overlap.

Quantitative PCR (qPCR) and RNA-seq

Total RNA was extracted using TRIzol reagent and then reverse-transcribed to cDNA using the ReverTraAce qPCR RT kit (Toyobo). Quantitative PCR was performed using SYBR Green (Vazyme Biotech) on the Light-Cycler Roche 480 (Roche). The mRNA expression was calculated using the equation $RQ=2^{-\Delta\Delta Ct}$ method and normalized to β-actin. The primer sequences are listed in Supplemental Table 6.

For RNA-sequencing analysis, a total of 3 μg RNA/sample was used as input for RNA sample preparation. Sequencing library were constructed using the NEBNext® UltraTM RNA Library Prep Kit (NEB) for Illumina according to the manufacturer's instructions, and index codes were added to attribute sequences to each sample. The clustering of the index-coded samples was performed on a cBot Cluster Generation System using TruSeq PE Cluster Kit v3-cBot-HS (Illumina) following the manufacturer's recommendations. After clustering, the library preparations were sequenced using an Illumina Novaseq platform (150bp paired-end). Sequenced raw reads were processed to obtain clean reads, and then mapped to the mouse genome (mm10) and differential gene expression analysis was performed using DESeq2 package (1.20.0). Library preparation, clustering, and sequencing were done by Novogene Co., Ltd. The GEO number of RNA-seq is GSE206868.

Colon explant culture and ELISA

Approximately 1cm of distal colon tissues was obtained longitudinally, weighted, washed three times with ice-cold PBS, and cultured in 1 ml RPMI 1640 (per 50 mg tissue) supplemented with 10% FBS and 20% penicillin/streptomycin for 24 h. The supernatant were collected and centrifuged to remove floating tissue debris. The concentrations of IL-1β, IL-6, and TNF-α in the supernatant were detected using mouse ELISA kits according to manufacturer's protocols (Invitrogen, 88-7013-88, 88-7064-88, 88-7324-88).

Immunofluorescence staining and TUNEL assay

Mouse colon tissues were fixed in 4% formalin, paraffin-embedded and cut into 4 μm sections. For immunofluorescence staining, sections were deparaffinized, rehydrated followed by antigen-retrieval in citric acid (10 mM, pH 6.0), and stained with primary antibodies including Gab1 (Abcam, ab59362, 1:200), CD45 (Proteintech, 60287-1-Ig, 1:200) , F4/80 (CST, 30325, 1:500) and Ly6G (Proteintech, 65140-1-Ig, 1:200) . To assess intestinal epithelial cell death, tissue was processed as mentioned above, and TUNEL assay was performed with In Situ Cell Death Kit (Roche) according to the manufacturer's recommendations. Sections were then stained with DAPI and analyzed by a confocal microscope.

Immunofluorescence staining and confocal microscopy

For tissue immunofluorescence staining, paraffin-embedded slides were deparaffinized and permeabilized with 0.5% Triton X100, and blocked with 5% goat serum for 1 h. Then the sections were stained with the primary antibodies against Gab1 (Abcam, ab59362), ZO-1 (ThermoFisher Scientific, 33-9100), CD45 (Proteintech, 60287-1-Ig), F4/80 (CST, 30325), Ly6G (CST, 31469) overnight, followed by secondary antibodies conjugated to Alexa fluor 488 or 594 (ThermoFisher Scientific), and cell nuclei was stained with DAPI. For cell immunofluorescence staining, HT29 cells were plated onto coverslips in 24 well

plates overnight, followed by T/S/Z stimulation for 3 h and PI staining (ThermoFisher Scientific) for 30min at 37 °C. Samples were fixed with 4% PFA, permeabilized with 0.5% Triton X100, and cell nuclei was stained with DAPI. The immunofluorescence images of stained cells or tissues were acquired using a confocal microscope (Olympus FluoView FV1000). For mouse intestine organoids staining, the organoids were plated in a matrigel-coated glass-bottom dish overnight and stimulated with necrotic signal for 8 h, and stained with PI for 30min at 37 °C. Live cell imaging was performed using a confocal microscope (FV3000, Olympus). The images were further processed using Olympus FluoView FV31S-SW and quantified by ImageJ.

FITC-dextran permeability assay

Untreated or DSS-treated mice were fasted for 4 h and then gavaged with FITC-dextran (MW 4,000 Da, 0.6 mg/g body weight, Sigma-Aldrich) dissolved in PBS. 4 h after gavage, mouse blood was collected and serum FITC-dextran levels were measured by Varioskan Flash fluorescence spectrophotometer (ThermoFisher Scientific, excitation of 488 nm and emission of 520 nm).

Transmission electron microscopy

HT29 cells were centrifuged and processed at a size of no bigger than 1 mm^3, fixed by 2.5% glutaraldehyde overnight at 4 °C. The samples were rinsed three times in PBS and then fixed in 1% osmium tetroxide for 60 min at room temperature. The samples were rinsed three times in PBS again, followed by 2% uranium acetate staining for 30 min. After that, the samples were dehydrated with a series of ethanol (30%, 50%, 70%, 80%, 90%, 95%, and 100%), embedded in an epoxy resin and processed for transmission electron microscopy according to standard procedures. Finally, the samples were examined by using an electron microscope (Tecnai G2 Spirit, Thermo FEI) operating at 120 kV.

ATP-based cell viability assay

Cell viability of HT29 cells upon T/S/Z stimulation was detected by using CellTiter-Glo® Luminescent Cell Viability Assay kit (Promega, G7571), in which based on quantitation of the cellular ATP to represent the presence of metabolically active cells. luminescent signal was recorded using Varioskan Flash fluorescence spectrophotometer (Thermo Scientific).

Western blot

Cells or colon homogenates from mice were lysed in RIPA lysis buffer (Beyotime) containing Complete Protease Inhibitor Cocktail and PhosSTOP phosphatase inhibitor (Roche). Protein concentration was quantified by BCA Protein Assay kit (Beyotime) and then denatured in SDS loading buffer. Proteins were separated using SDS-PAGE gels and transferred to nitrocellulose membranes (Pall, Port Washington). The primary antibodies used for Western blot were Gab1 (Proteintech, 26200-1-AP, 1:1000), Gab2 (Abcam, ab235932, 1:1000) , Shp2 (CST, 3397, 1:1000), human-p-RIPK1 (S166) (CST, 65746, 1:500), RIPK1 (CST, 3493, 1:1000), human-p-RIPK3 (S227) (CST, 93654, 1:1000), human-RIPK3 (CST, 13526, 1:1000), human-p-MLKL (S358) (CST, 91689, 1:1000), human-MLKL (CST, 14993), mouse-p-RIPK1 (S166) (CST, 53286, 1:500), mouse-p-RIPK3 (T231/S232) (CST, 91702, 1:500), mouse-RIPK3 (CST, 15828, 1:1000), mouse-p-MLKL (S345) (CST, 37333, 1:500), mouse-MLKL (CST, 37705, 1:1000), HMGB1 (Abcam, ab79823, 1:1000), GSDMD (Abcam, ab209845), p-Stat3 (Y705) (CST, 9145, 1:1000), Stat3 (CST, 12640, 1:1000), p53 (CST, 2524, 1:1000), cl-caspase3 (CST, 9664, 1:1000), Bcl-2 (Proteintech, 12789-1-AP, 1:1000), Bcl-XL (Proteintech, 10783-1-AP, 1:1000), Bax (Proteintech, 50599-2-Ig, 1:1000), ACSL4 (Abcam, ab155282), FTH1 (Abcam, ab183781), GPX4 (Santa Cruz, SC-166570), β-actin (Huabio, M1210-2, 1:2000), followed by IRDye 680/800 secondary antibodies (LI-COR).

Co-immunoprecipitation analysis

Cells were collected and lysed in NP-40 lysis buffer (Beyotime) containing Complete Protease Inhibitor Cocktail and PhosSTOP phosphatase inhibitor at 4 °C. Protein A/G Dynabeads (Bio-Rad) were pretreated with primary antibody for 10 min at 25 °C and then washed with PBST for five times. The antibody-conjugated magnetic beads were then incubated with cleared cell lysates overnight at 4 °C. The immunoprecipitates were washed and eluted with 1 × SDS loading buffer followed by Western blot analysis.

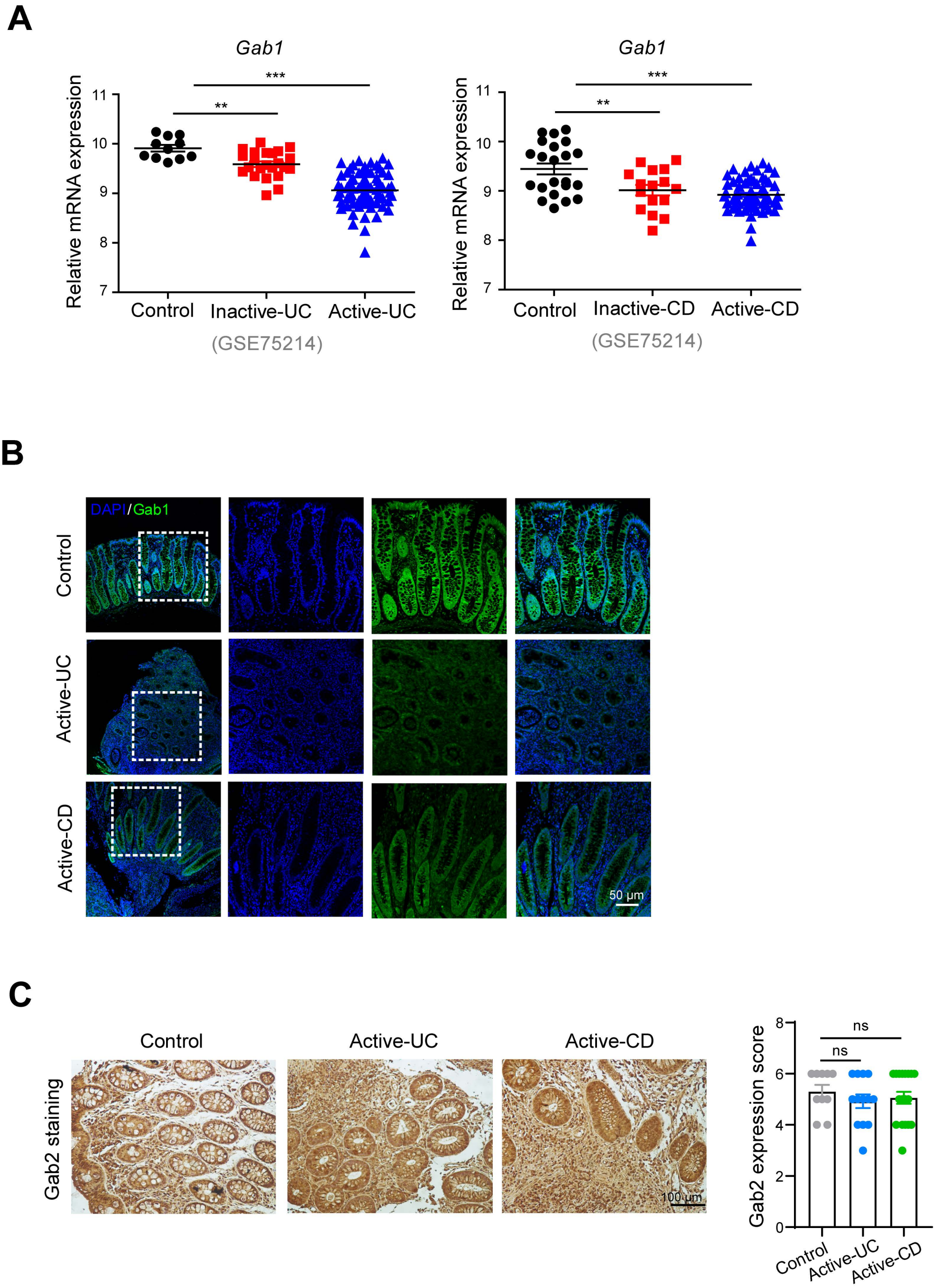

A
Gab1
Relative mRNA expression
Control
Inactive-UC
Active-UC
(GSE75214)
Gab1
Relative mRNA expression
Control
Inactive-CD
Active-CD
(GSE75214)
B
DAPI/Gab1
Control
Active-UC
Active-CD
50 μm
C
Control
Active-UC
Active-CD
Gab2 staining
100 μm
Gab2 expression score
ns
ns

Figure S1. Gab1 expression is decreased in IBD patients whereas Gab2 remains unaltered.

(A) Statistical analysis of *Gab1* mRNA expression in colonic biopsies from patients with inactive UC (n = 23), active UC (n = 74) and matched normal controls (n = 11), as well as patients with inactive CD (n = 16), active CD (n = 59) and matched normal controls (n = 22). Data were collected from GEO database GSE75214.

(B) Immunofluorescence staining for Gab1 (green) and DAPI (blue) in colonic biopsies from normal controls and patients with active UC or active CD. Scale bars, 50 μm.

(C) Representative IHC staining of Gab2 in colonic mucosa from patients with active UC (n = 13) or active CD (n = 18), and normal controls (n = 10). Scale bars, 100 μm.

Data were presented as mean ± SEM. Statistical significance was assessed by one-way ANOVA with multiple comparisons test (A and C); $^{**}p < 0.01$, $^{***}p < 0.001$; ns, not significant.

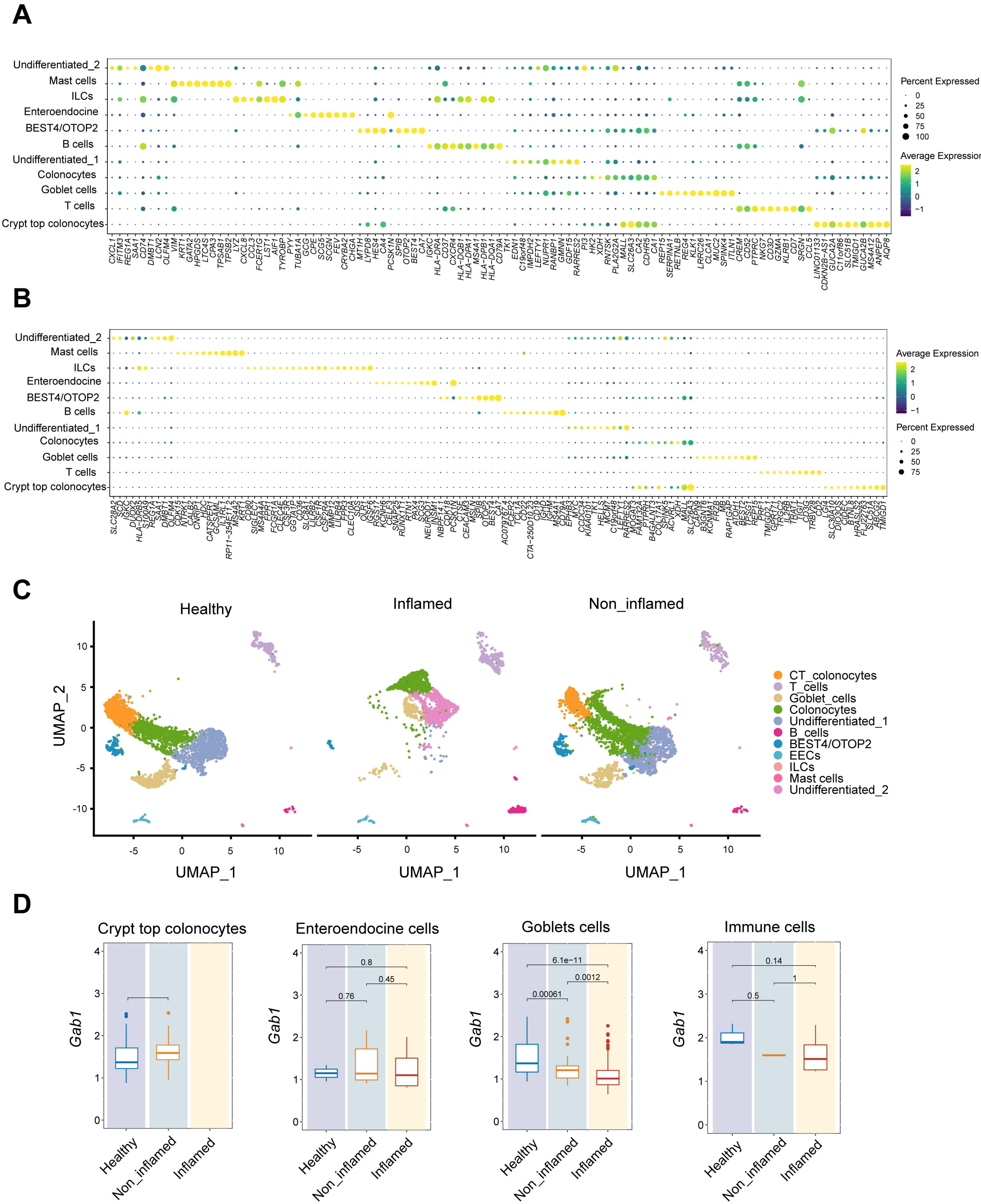
A
Undifferentiated_2
Mast cells
ILCs
Enteroendocine
BEST4/OTOP2
B cells
Undifferentiated_1
Colonocytes
Goblet cells
T cells
Crypt top colonocytes
Percent Expressed
0
25
50
75
100
Average Expression
2
1
0
−1
B
Undifferentiated_2
Mast cells
ILCs
Enteroendocine
BEST4/OTOP2
B cells
Undifferentiated_1
Colonocytes
Goblet cells
T cells
Crypt top colonocytes
Average Expression
2
1
0
−1
Percent Expressed
0
25
50
75
C
Healthy
Inflamed
Non_inflamed
UMAP_2
UMAP_1
10
5
0
-5
-10
CT_colonocytes
T_cells
Goblet_cells
Colonocytes
Undifferentiated_1
B_cells
BEST4/OTOP2
EECs
ILCs
Mast cells
Undifferentiated_2
D
Crypt top colonocytes
Enteroendocine cells
Goblets cells
Immune cells
Gab1
0.8
0.45
0.76
6.1e−11
0.0012
0.00061
0.14
1
0.5
Healthy
Non_inflamed
Inflamed

Figure S2. Cell identification of scRNA-seq analysis in colon crypts from UC patients and healthy controls. The data were obtained from GEO database GSE116222.

(A-B) Heat map showing key differentially expressed genes between cell clusters in colon crypts. The colour indicates a fold change (expressed in log_2) and the point size shows the confidence interval for the observation.

(C) UMAP plot of single-cell clusters in ulcerative colitis and healthy controls. n = 3 per group.

(D) Box blot showing relative *Gab1* expression in epithelial-cell subpopulations and immune cells in patients with inflamed/non-inflamed UC and healthy controls. n = 3 per group.

Data were shown as mean ± SEM. Wilcox test was used to determine statistical significance.

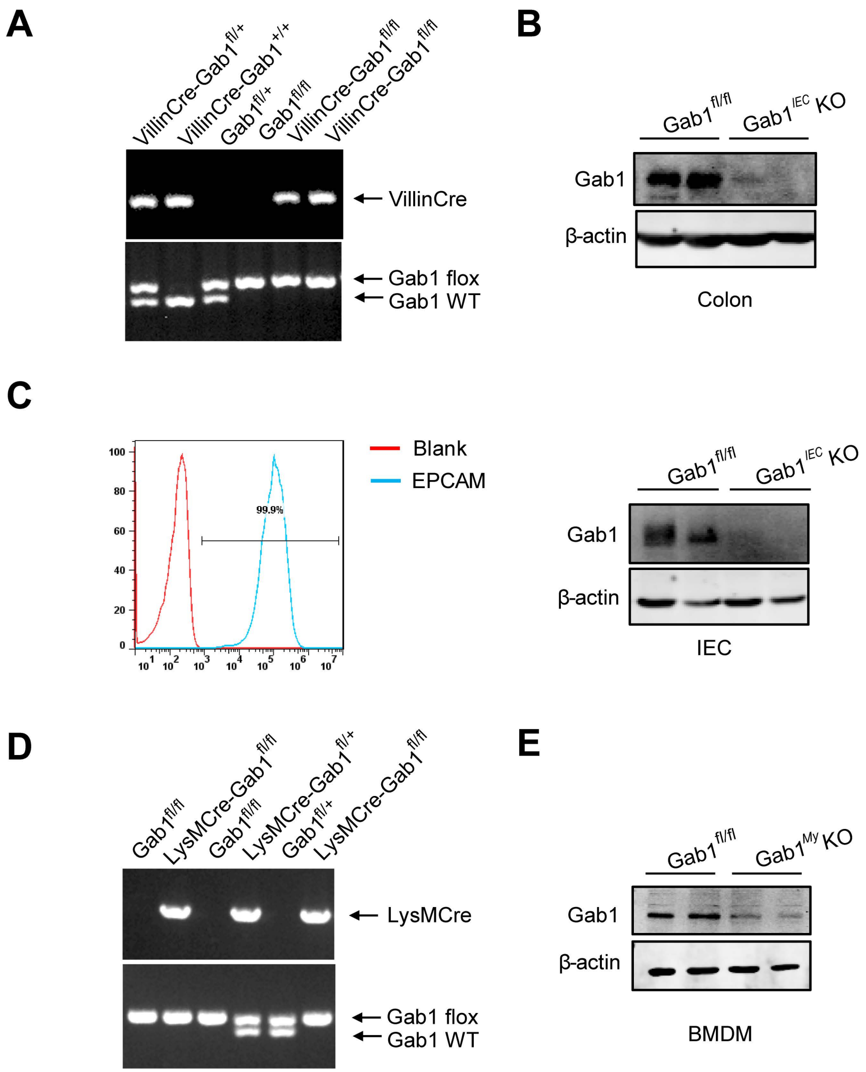

A
VillinCre-Gab1fl/+
VillinCre-Gab1+/+
Gab1fl/+
Gab1fl/fl
VillinCre-Gab1fl/fl
VillinCre-Gab1fl/fl
VillinCre
Gab1 flox
Gab1 WT
B
Gab1fl/fl
Gab1IEC KO
Gab1
β-actin
Colon
C
Blank
EPCAM
99.9%
Gab1fl/fl
Gab1IEC KO
Gab1
β-actin
IEC
D
Gab1fl/fl
LysMCre-Gab1fl/fl
Gab1fl/fl
LysMCre-Gab1fl/+
Gab1fl/+
LysMCre-Gab1fl/fl
LysMCre
Gab1 flox
Gab1 WT
E
Gab1fl/fl
Gab1My KO
Gab1
β-actin
BMDM

Figure S3. The generation of Gab1^{IEC} KO and Gab1^{My} KO mice.

(A) Genotyping analysis of VillinCre-driven IEC conditional knockout mice was performed with mouse tail genomic DNA by PCR.

(B) Western blotting of Gab1 level in colon tissues of Gab1$^{fl/fl}$ and Gab1^{IEC} KO mice.

(C) Western blotting of Gab1 level in EPCAM^{+} IECs from Gab1$^{fl/fl}$ and Gab1^{IEC} KO mice.

(D) Genotyping analysis of LysMCre-driven myleoid conditional knockout mice was performed with mouse tail genomic DNA by PCR.

(E) Immunoblotting of Gab1 in BMDMs from Gab1$^{fl/fl}$ and Gab1^{My} KO mice.

Data were representative of at least three independent experiments. BMDM, Bone marrow derived macrophage;

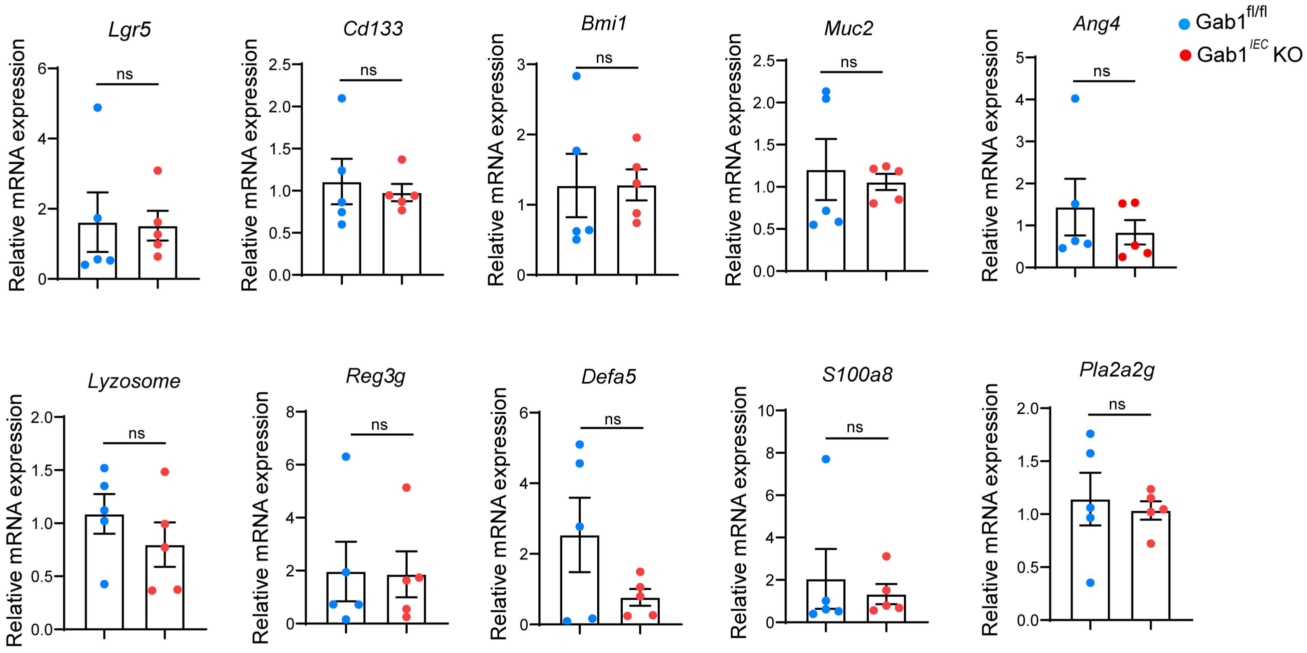

Lgr5
Cd133
Bmi1
Muc2
Ang4
Gab1fl/fl
Gab1IEC KO
Lyzosome
Reg3g
Defa5
S100a8
Pla2a2g
Relative mRNA expression
ns

Figure S4. No significant difference was found in mRNA expression of stem cell markers, antimicrobial peptides and mucins in epithelial Gab1-deficient mice

Quantitative PCR of stem cell markers (*Lgr5, Cd133, Bmi1*), antimicrobial peptides (*Ang4, Lysozyme, Reg3g, Defa5, S100a8*, *Pla2g2a*) and mucins (*Muc2*) of intestine from Gab1^{IEC} KO mice and littermate controls. n = 5 per group.

Data were presented as mean ± SEM. Statistical significance was generated by using two-tailed Student’s t-test; ns, not significant.

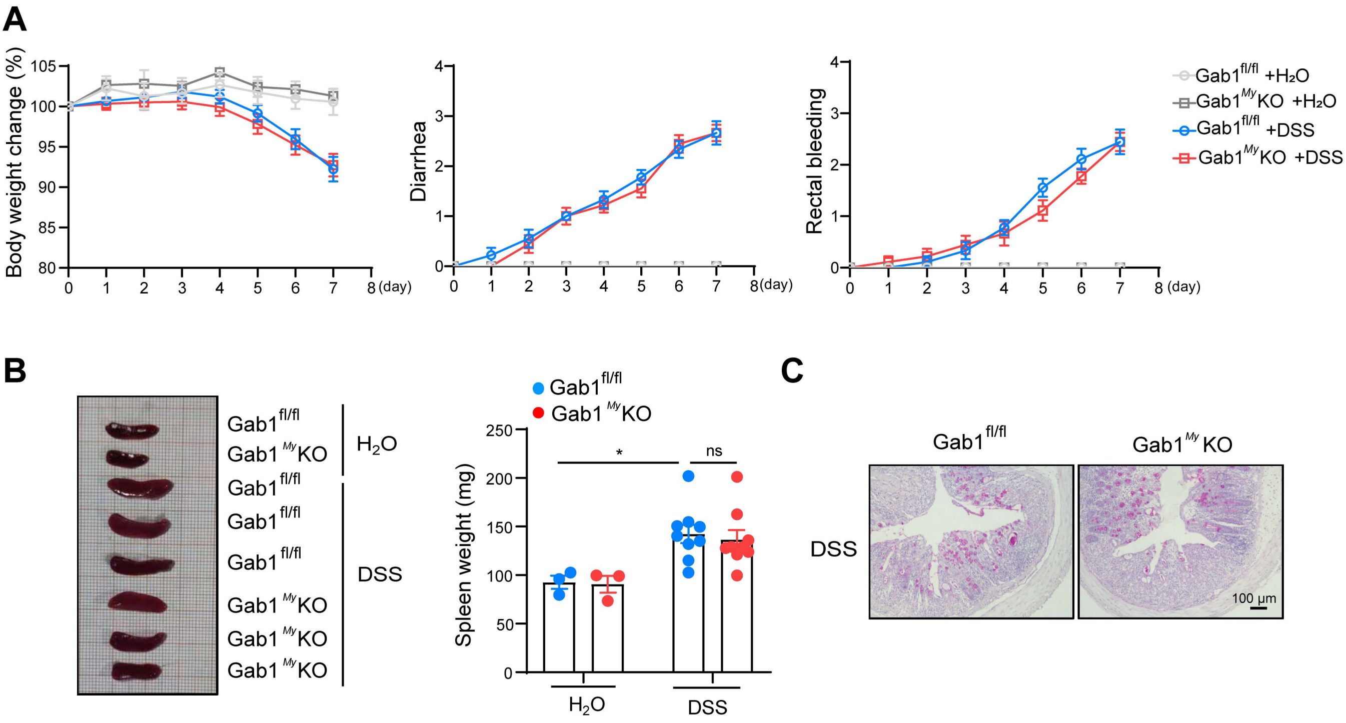

A
Body weight change (%)
Diarrhea
Rectal bleeding
(day)
Gab1fl/fl +H2O
Gab1MyKO +H2O
Gab1fl/fl +DSS
Gab1MyKO +DSS
B
Gab1fl/fl
Gab1 MyKO
H2O
DSS
Spleen weight (mg)
*
ns
C
DSS
100 μm

Figure S5. Knockout of Gab1 in myleoid did not render the mice susceptible to colitis. Gab1$^{fl/fl}$ and Gab1^{My} KO mice were administrated with water (n=3) or 3% DSS (n=9) for 7 days to induce experimental colitis.

(A) Body weight loss, diarrhea and rectal bleeding Gab1$^{fl/fl}$ or Gab1^{My} KO mice were assessed daily.

(B) Gross morphology images of spleen and spleen weight of Gab1$^{fl/fl}$ or Gab1^{My} KO mice.

(C) Representative PAS staining of colonic sections from Gab1^{My} KO mice and littermate controls. Scale bars, 100 μm.

Data were presented as mean ± SEM. Statistical significance was assessed by using two-way ANOVA with multiple comparisons test; *$p<0.05$; ns, not significant.

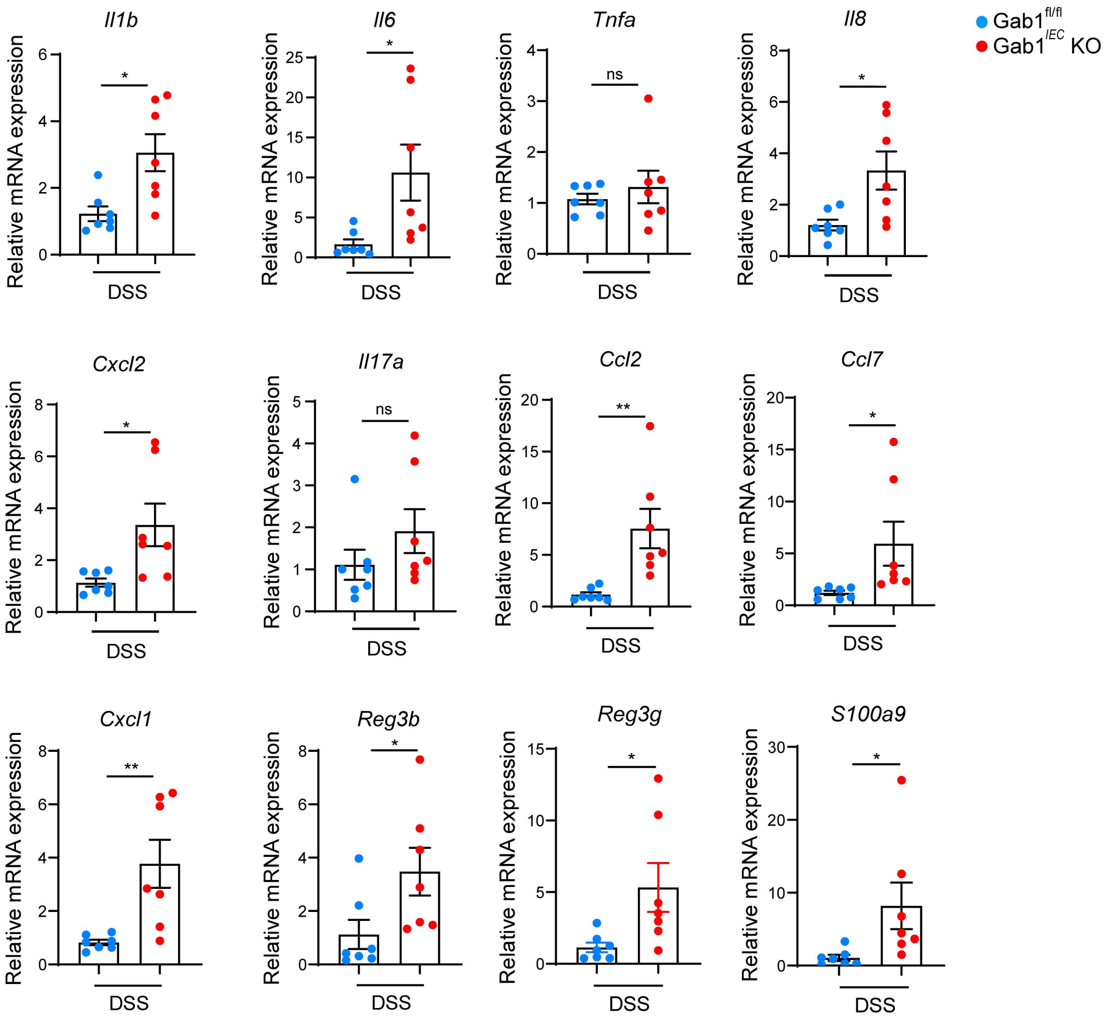

Gab1fl/fl
Gab1IEC KO
Il1b
Il6
Tnfa
Il8
Cxcl2
Il17a
Ccl2
Ccl7
Cxcl1
Reg3b
Reg3g
S100a9
Relative mRNA expression
DSS
ns
*
**

Figure S6. Validation for the expression of proinflammatory cytokines, chemokines and antimicrobial peptides in DSS-challenged colons.

Quantitative real-time PCR validation for the mRNA expression of proinflammatory cytokines (*Il1b, Il6, TNFa, Il8* and *Il17a*), chemokines (*Cxcl1*, *Cxcl2, Ccl2* and *Ccl7*), as well as antimicrobial peptides (*Reg3b, Reg3g* and *S100a9*) in colonic tissue from $Gab1^{fl/fl}$ and $Gab1^{IEC}$ KO mice. n=7 per group. Data were represented as mean ± SEM. Statistical significance was assessed by using two-tailed Student's t-test; *p<0.05, ** p<0.01; ns, not significant.

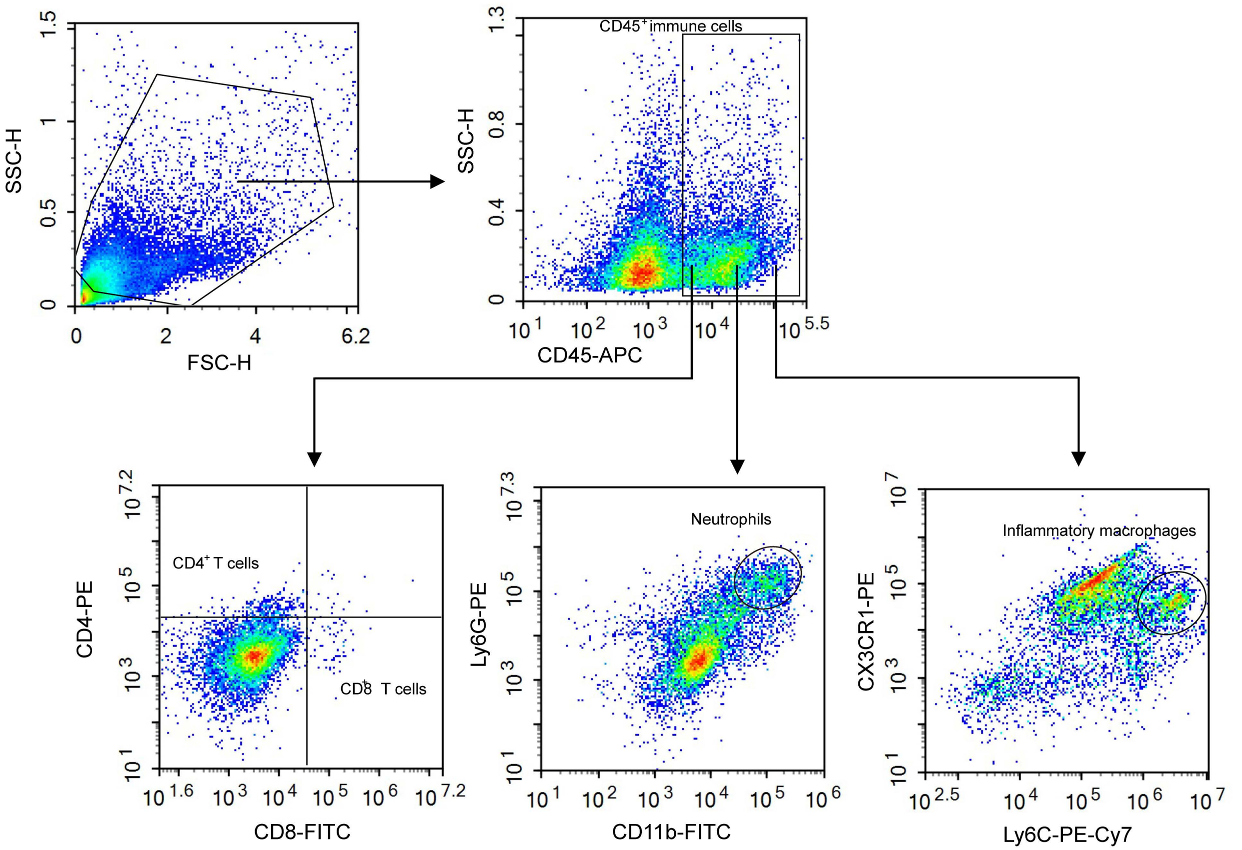
SSC-H
FSC-H
CD45+ immune cells
SSC-H
CD45-APC
CD4-PE
CD4+ T cells
CD8+ T cells
CD8-FITC
Ly6G-PE
Neutrophils
CD11b-FITC
CX3CR1-PE
Inflammatory macrophages
Ly6C-PE-Cy7

Figure S7. Gating strategy for flow cytometry analysis.

Gating strategy for identification of $CD45^+$ immune cells, neutrophils, T cells and inflammatory macrophages.

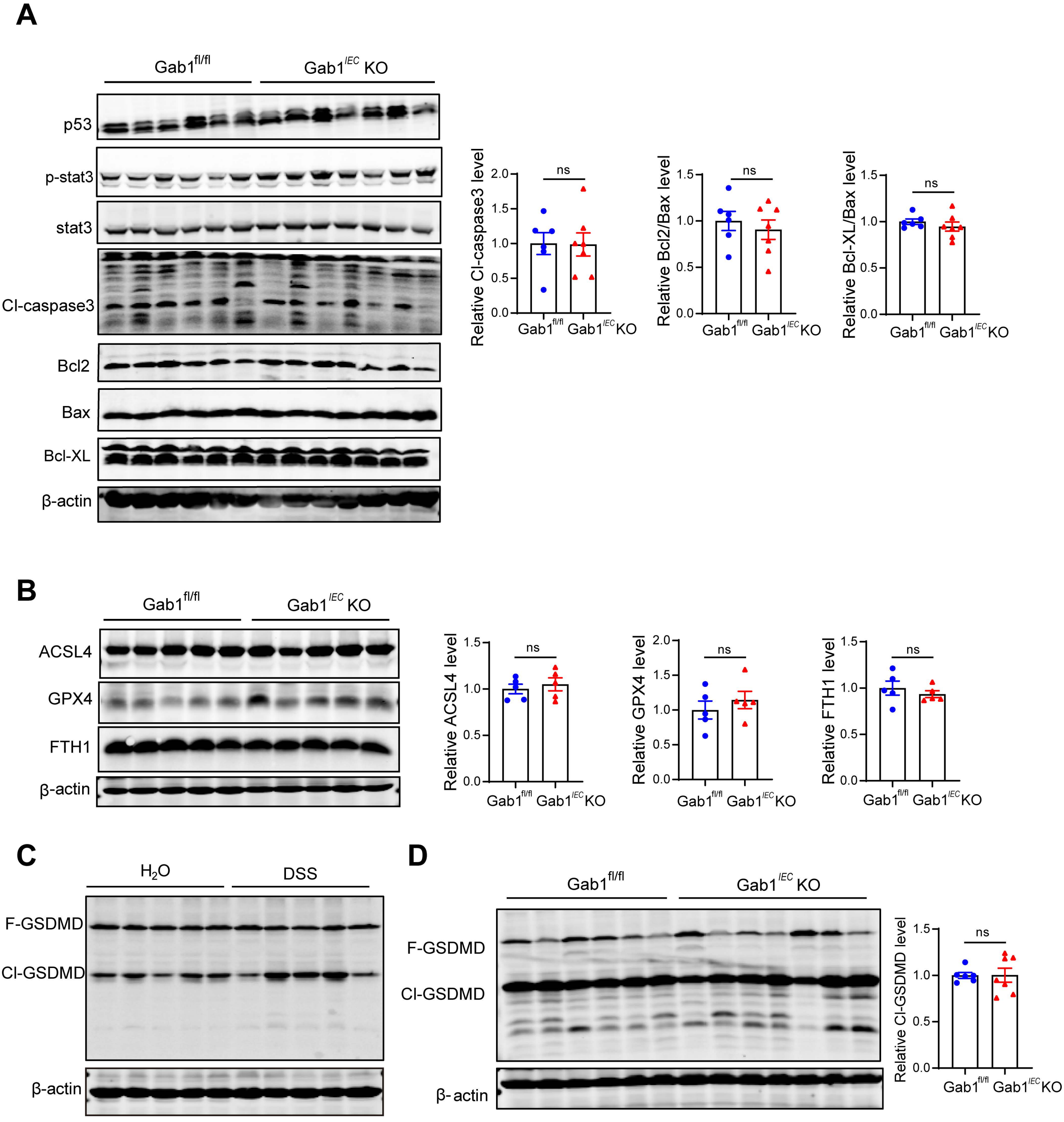
A
Gab1fl/fl
Gab1IEC KO
p53
p-stat3
stat3
Cl-caspase3
Bcl2
Bax
Bcl-XL
β-actin
Relative Cl-caspase3 level
Relative Bcl2/Bax level
Relative Bcl-XL/Bax level
ns
Gab1fl/fl Gab1IEC KO
B
ACSL4
GPX4
FTH1
Relative ACSL4 level
Relative GPX4 level
Relative FTH1 level
C
H2O
DSS
F-GSDMD
Cl-GSDMD
D
Relative Cl-GSDMD level

Figure S8. The minimal role of apoptosis, ferroptosis and pyroptosis in contributing to the pathology of Gab1^{IEC} KO mice in colitis.

(A) Immunoblot analysis of apoptosis executioner cleaved-caspase 3 and apoptosis-related proteins (including p53, Bcl2, Bax, Bcl-XL) in colonic protein from DSS-treated mice on day 7. n=6, 7, respectively.

(B) Immunoblot analysis of ferroptosis-related proteins including ACSL4, GPX4 and FTH1 in colonic protein from DSS-treated mice on day 7. n=5 for each group.

(C-D) Immunoblot analysis of cleaved-GSDMD, the pore-forming protein of pyroptosis, in colonic protein from DSS-treated mice on day 7. n=5 for each group (C) and n=6, 7, respectively (D).

Quantitative data were presented as mean ± SEM. Statistical significance was assessed by using two-tailed Student’s t-test. ns, not significant.

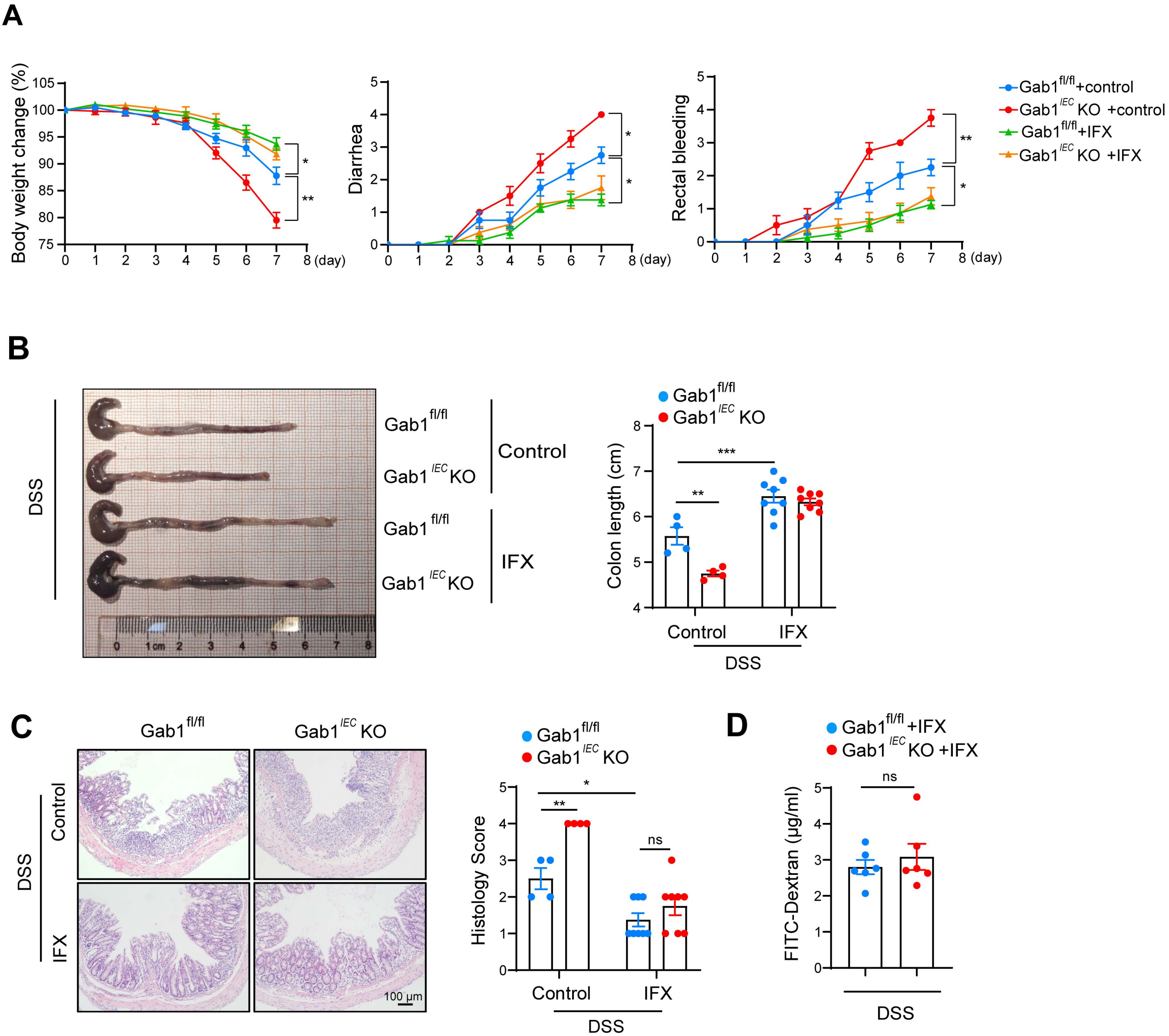

A
Body weight change (%)
Diarrhea
Rectal bleeding
(day)
Gab1fl/fl+control
Gab1IEC KO +control
Gab1fl/fl+IFX
Gab1IEC KO +IFX
B
DSS
Gab1fl/fl
Gab1IEC KO
Control
IFX
Colon length (cm)
C
Histology Score
100 µm
ns
D
Gab1fl/fl +IFX
Gab1IEC KO +IFX
FITC-Dextran (µg/ml)

Figure S9. TNF neutralization rescued epithelial Gab1-deficient mice from DSS-induced colitis. Gab1$^{fl/fl}$ and Gab1^{IEC} KO mice were exposed to 3% DSS in drinking water as previously described and received either saline or IFX intraperitoneally at a dose of 0.0615 mg/g body weight on day 3.

(A) Relative percent change in body weight, diarrhea and rectal bleeding scores were monitored daily. n = 4, 4, 8, 8, respectively.

(B) Gross morphology images of the colon from Gab1$^{\mathrm{fl/fl}}$ and Gab1^{IEC} KO mice with different treatment. Colon length were measured on day 7. n = 4, 4, 8, 8, respectively.

(C) Representative H&E-stained images and histological scores of the distal colon were assessed on day 7. Scale bars, 100 μm. n = 4, 4, 8, 8, respectively.

(D) Mice were administrated with FITC-dextran intragastrically 4 hours before sacrifice, then serum FITC-dextran level was examined by fluorescence spectrophotometer. n=6 for each group.

Data were presented as mean ± SEM. Statistical significance was assessed by using two-way ANOVA with multiple comparisons test (A-C) and two-tailed Student's t-test (D); *p<0.05, **p<0.01, *** p<0.001; ns, not significant.

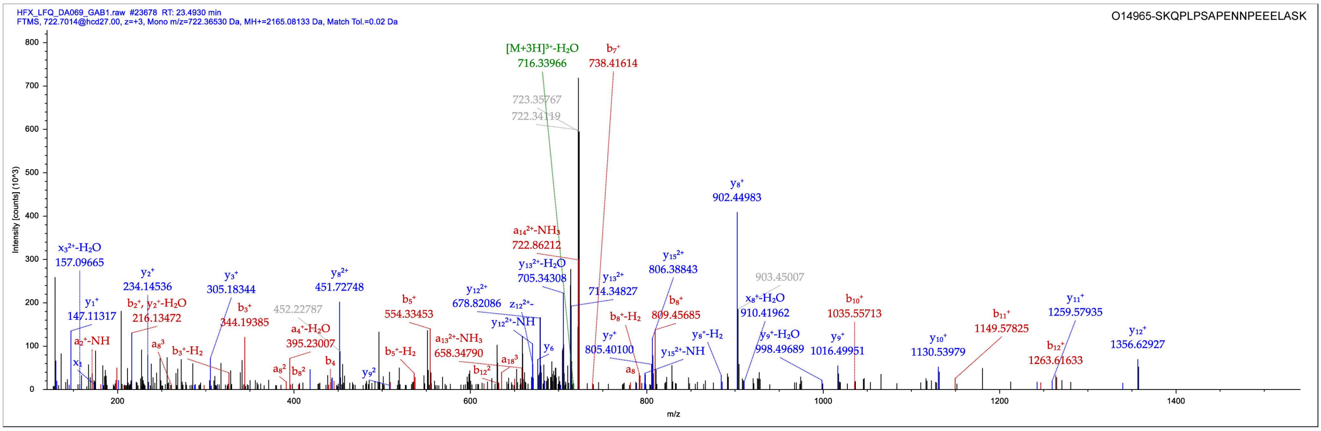
HFX_LFQ_DA069_GAB1.raw #23678 RT: 23.4930 min
FTMS, 722.7014@hcd27.00, z=+3, Mono m/z=722.36530 Da, MH+=2165.08133 Da, Match Tol.=0.02 Da
O14965-SKQPLPSAPENNPEEELASK
Intensity [counts] (10^3)
m/z
[M+3H]³⁺-H₂O 716.33966
b₇⁺ 738.41614
723.35767
722.34119
y₈⁺ 902.44983
a₁₄²⁺-NH₃ 722.86212
x₃²⁺-H₂O 157.09665
y₁₃²⁺-H₂O 705.34308
y₁₅²⁺ 806.38843
903.45007
y₂⁺ 234.14536
y₃⁺ 305.18344
y₈²⁺ 451.72748
y₁₃²⁺ 714.34827
y₁⁺ 147.11317
b₂⁺, y₂⁺-H₂O 216.13472
b₃⁺ 344.19385
452.22787
b₅⁺ 554.33453
y₁₂²⁺ 678.82086
z₁₂²⁺-
b₈⁺ 809.45685
x₈⁺-H₂O 910.41962
b₁₀⁺ 1035.55713
b₁₁⁺ 1149.57825
y₁₁⁺ 1259.57935
b₈⁺-H₂
y₁₂²⁺-NH
a₂⁺-NH
a₄⁺-H₂O 395.23007
a₁₃²⁺-NH₃ 658.34790
y₇⁺ 805.40100
y₈⁺-H₂
y₉⁺-H₂O 998.49689
y₉⁺ 1016.49951
y₁₀⁺ 1130.53979
y₁₂⁺ 1356.62927
a₈³
b₃⁺-H₂
y₆
y₁₅²⁺-NH
b₁₂⁺ 1263.61633
x₁
b₄
b₅⁺-H₂
a₁₈³
a₈
a₈²
b₈²
y₉²
b₁₂²
800
700
600
500
400
300
200
100
0
200
400
600
800
1000
1200
1400

Figure S10. Mass spectrum of the AURKA peptides from Gab1 co-immunoprecipitate.

Lysates of HEK293T cells were immunoprecipitated with anti-Gab1 antibody, and then subjected to MS analysis.

Supplemental Table 1. Basic Information of Clinical Samples

**Characteristics**	HS (n=10)	UC (n=19)	CD (n=24)
Age, Year (mean ± SD)	45.90 ± 5.92	37.84 ± 11.36	26.96 ± 8.36
Male / Female n (%)	4 (40.0%) / 6 (60.0%)	10 (52.6%) / 9 (47.4%)	16 (66.7%) / 8 (33.3%)
**Montreal classification**			
n (%)	10 (100%)	19 (44.2%)	24 (55.8%)
A1 / A2 / A3 (n)	NA	NA	3 / 17 / 4
L1 / L2 / L3 / L4 (n)	NA	NA	3 / 2 / 19 / 0
B1 / B2 / B3 (n)	NA	NA	17 / 3 / 4
E1 / E2 / E3 (n)	NA	0 / 4 / 15	NA
SES-CD (mean ± SD)	NA	NA	17.42 ±6.05
MS (mean ± SD)	NA	9.39 ± 2.37	NA
**Biopsy location**			
Ileum / Colon (n)	2 / 8	0 / 19	10 / 14
**Disease stage**			
Mild / Severe	NA	6 / 13	7 / 17
**CRP** (mean ± SD)	0.69 ±0.50	28.92±18.77	33.37±19.60
**Previous therapy**			
5-aminosalicylates	NA	12	11
Salazosulfapyridine	NA	0	2
Azathioprine	NA	0	0
Glucocorticoids	NA	2	2
Methotrexate	NA	0	0
Biologics	NA	0	0

HS: Health sample. UC: ulcerative colitis. CD: Crohn's disease. NA: not-available. Montreal classification of extent of UC: E1, ulcerative proctitis; E2, left sided UC; E3, extensive UC. Age at diagnosis by Montreal classification for CD: A1, ≤16; A2, 17-40; A3, >40. Localization of disease by Montreal classification: L1, ileal; L2, colonic; L3: ileocolonic; L4: upper gastrointestinal tract. Disease behavior for Montreal classification for CD: B1, non-stricturing; B2, stricturing; B3, penetrating. SES-CD, Simplified Endoscopic Score for Crohn's Disease; MS, Mayo score. CRP, C-reactive protein.

Supplemental Table 2. Basic Information of GSE75214

Characteristics	Colon					Ileum		
	UC		CD		Controls	CD		Controls
	Active (n=74)	Inactive (n=23)	Active (n=8)	Inactive (n=26)	(n=11)	Active (n=51)	Inactive (n=16)	(n=11)
**Male/Female (%)**	43/31 (58/42)	12/11 (52/48)	2/6 (25/75)	14/12 (54/46)	5/6 (45/55)	20/31 (39/61)	6/10 (38/62)	6/5 (55/45)
**Median (IQR) age (years)**	45 (32-54)	43 (29-56)	38 (34-44)	38 (26-50)	68 (62-73)	41 (29-54)	43 (26-52)	59 (52-73)
**UC disease extent**								
UC Left-sided colitis (%)	35 (47)	13 (57)	NA	NA	NA	NA	NA	NA
Pancolitis (%)	39 (53)	10 (44)	NA	NA	NA	NA	NA	NA
**CD disease location**								
Ileocolon (%)	NA	NA	3 (38)	8 (31)	NA	43 (84)	10 (63)	NA
Ileum (%)	NA	NA	0 (0)	8 (31)	NA	8 (16)	6 (38)	NA
Colon (%)	NA	NA	5 (63)	10 (38)	NA	0 (0)	0 (0)	NA
**Medication use**								
5-Aminosalicylates (%)	59 (80)	22 (96)	1 (13)	7 (27)	NA	11 (22)	3 (19)	NA
Corticosteroids (%)	31 (42)	2 (9)	2 (25)	5 (19)	NA	8 (16)	0 (0)	NA
Azathioprine/6-Mercaptopurine (%)	13 (18)	10 (44)	0 (0)	12 (46)	NA	8 (16)	1 (6)	NA
Methotrexate (%)	2 (3)	0 (0)	0 (0)	0 (0)	NA	1 (2)	0 (0)	NA
Anti-TNF (%)	0 (0)	11 (48)	0 (0)	11 (42)	NA	1 (2)	1 (6)	NA

UC, ulcerative colitis; CD, Crohn's disease; n, number; IQR, interquartile range; NA, non-applicable.

Supplemental Table 3. Basic Information of GSE92415

Characteristics	HS	UC		
	(n=21)	Baseline (n=87)	Treatment （n=75）	
Placebo / Golimumab n (%)	NA	NA	Placebo 25 (33.3%)	Golimumab 50 (66.7%)
Age, Year (mean ± SD)	NA	42.08±11.72	38.68±10.44	44.1±12.3
**Biopsy location**				
Ileum / Colon (n)	0 / 21	0 / 87	0 / 25	0 / 50
Mayo Score	NA	8.38±1.38	5.84±2.20	5.2±2.5
Clinical response n (%)	NA	NA	NA	29 (58%)

HS: Health sample. UC: ulcerative colitis. NA: not-available.

Supplemental Table 4. Basic Information of GSE116222

**Characteristics**	HS (n=3)	UC (n=3)
Age, Year (median [Range])	50 [47-74]	55 [36-78]
Gender (n (%) male)	1 (33%)	2 (66%)

HS: Health sample. UC: ulcerative colitis. NA: not-available.

Supplemental Table 5. Basic Information of GSE16879

**Characteristics**	Control (n=12)	UC (n=24)	CDc (n=19)	CDi (n=18)
Age, Year (Median, IQR)	NA	41.4 (31.9-50.9)	31.8 (23.7-47.5)	46.4 (34-55.3)
Male / Female n (%)	NA	14 / 10<br>(58.3/ 41.7)	11 / 8<br>(57.9 /42.1)	9 / 9<br>(50 / 50)
**Biopsy location**				
Ileum / Colon (n)	6 / 6	0 / 24	0 / 19	18 / 0
**CRP at first IFX** (Median, IQR)	0.69 ±0.50	4 (1.8–19.1)	10.2 (4.3–35)	7.4 (2.3–10.9)
**Previous therapy** (%)				
5-aminosalicylates	NA	18 (75)	8 (42.1)	5 (27.8)
Corticosteroids	NA	7 (29.2)	4 (21.1)	2 (11.1)
Azathioprine	NA	15 (62.5)	14 (73.7)	7 (38.9)
Methotrexate	NA	0 (0)	0 (0)	0 (0)
Corticosteroids with immunosuppressants	NA	3 (12.5)	2 (10.5)	1 (6)
Clinical response n (%)	NA	8 (33.3)	11 (57.9)	8 (44.4)

UC: ulcerative colitis. CD: Crohn’s disease. NA: not-available. CRP, C-reactive protein.

Supplemental Table 6. RT-qPCR Primer Sequences

Gene	Forward Primer (5’ to 3’)	Reverse Primer (5’ to 3’)
**Mouse Genes**		
Lgr5	GGACCAGATGCGATACCGC	CAGAGGCGATGTAGGAGACTG
Bmi-1	AAATCCCCACTTAATGTGTGTCC	CTTGCTGGTCTCCAAGTAACG
Cd133	ACTGGGGCTGTGTGGAAAG	GCATTGAAGGTATCTTGGGTCTC
Muc2	ATGCCCACCTCCTCAAAGAC	GTAGTTTCCGTTGGAACAGTGAA
Ang4	GGTTGTGATTCCTCCAACTCTG	CTGAAGTTTTCTCCATAAGGGCT
Lyz1	GGAATGGATGGCTACCGTGG	CATGCCACCCATGCTCGAAT
Reg3b	TGGTTTGATGCAGAACTGGC	CCATTCCCATCCACCTCCAT
Reg3g	CCGTGCCTATGGCTCCTATTG	GCACAGACACAAGATGTCCTG
Defa5	TTGTCCTCCTCTCTGCCCTT	GACACAGCCTGGTCCTCTTC
S100a8	CTGAGTGTCCTCAGTTTGTG	TTGCATTGTCACTATTGATGTCC
S100a9	CACAGTTGGCAACCTTTATG	CAGCTGATTGTCCTGGTTTG
Pla2a2g	TGGCTCAATACAGGACCAAGG	GTGGCATCCATAGAAGGCATAG
Il6	AGTTGCCTTCTTGGGACTGA	TCCACGATTTCCCAGAGAAC
Il8	CGCTTCTCTGTGCAGCGCTGCTGCT	AAGCCTCGCGACCATTCTTGAGTG
Il1a	CGAAGACTACAGTTCTGCCATT	GACGTTTCAGAGGTTCTCAGAG
Il1b	TGTGGCTGTGGAGAAGCTGT	CAGCTCATATGGGTCCGAGA
Il17a	GCTCCAGAAGGCCCTCAG	CTTTCCCTCCGCATTGACA
Tnfa	CTGGGACAGTGACCTGGCT	GCACCTCAGGGAAGAGTCTG
Cxcl1	CTGGGATTCACCTCAAGAACATC	CAGGGTCAAGGCAAGCCTC
Cxcl2	CCTGGTTCAGAAAATCATCCA	CTTCCGTTGAGGGACAGC
β-actin	AACAGTCCGCCTAGAAGCAC	CGTTGACATCCGTAAAGACC
**Human Genes**		
Gab1	CTACCTGTTGCTCATCAACTGT	GGGACGTTATCATTGCAGTCTG
Il-1β	CTCGCCAGTGAAATGATGGCT	GTCGGAGATTCGTAGCTGGAT
Cxcl1	CCCCAAGAACATCCAAAGTGT	TGGATTTGTCACTGTTCAGCA
Cxcl2	CACTCAAGAATGGGCAGAAAG	TCAGGAACAGCCACCAATAAG
Cxcl8	CAGTTTTGCCAAGGAGTGCT	ACTTCTCCACAACCCTCTGC
β-actin	AAGGTGAAGGTCGGAGTCAAC	GGGGTCATTGATGGCAACAATA
